# Body Composition and Metabolic Changes in a Lyon Hypertensive Congenic Rat and Identification of *Ercc6l2* as a Positional Candidate Gene

**DOI:** 10.3389/fgene.2022.903971

**Published:** 2022-06-24

**Authors:** Karen C. Clark, Valerie A. Wagner, Katie L. Holl, John J. Reho, Monika Tutaj, Jennifer R. Smith, Melinda R. Dwinell, Justin L. Grobe, Anne E. Kwitek

**Affiliations:** ^1^ Department of Physiology, Medical College of Wisconsin, Milwaukee, WI, United States; ^2^ Comprehensive Rodent Metabolic Phenotyping Core, Medical College of Wisconsin, Milwaukee, WI, United States; ^3^ Department of Biomedical Engineering, Medical College of Wisconsin, Milwaukee, WI, United States; ^4^ Rat Genome Database, Medical College of Wisconsin, Milwaukee, WI, United States; ^5^ Mellowes Center for Genomic Sciences and Precision Medicine, Medical College of Wisconsin, Milwaukee, WI, United States; ^6^ Cardiovascular Center, Medical College of Wisconsin, Milwaukee, WI, United States

**Keywords:** congenic rat, QTL mapping, rat model, body weight, metabolism, fat distribution, adipocyte hypertrophy, *ERCC6L2* gene

## Abstract

Central obesity is genetically complex, and its exponential increase in the last decades have made it a critical public health issue. The Lyon Hypertensive (LH) rat is a well-characterized hypertensive model that also exhibits spontaneous and profound differences in body weight and adiposity, relative to its metabolically healthy control, the Lyon Normotensive (LN) rat. The mechanisms underlying the body weight differences between these strains are not well-understood, thus a congenic model (LH^17^LNa) was developed where a portion of the proximal arm of LN chromosome 17 is introgressed on the LH genomic background to assess the contribution of LN alleles on obesity features. Male and female LH^17^LNa rats were studied, but male congenics did not significantly differ from LH in this study. Female LH^17^LNa rats exhibited decreases in total body growth, as well as major alterations to their body composition and adiposity. The LH^17^LNa female rats also showed decreases in metabolic rate, and a reduction in food intake. The increased adiposity in the female LH^17^LNa rats was specific to abdominal white adipose tissue, and this phenomenon was further explained by significant hypertrophy in those adipocytes, with no evidence of adipocyte hyperplasia. Sequencing of the parental strains identified a novel frameshift mutation in the candidate gene *Ercc6l2*, which is involved in transcription-coupled DNA repair, and is implicated in premature aging. The discovery of the significance of Ercc6l2 in the context of female-specific adipocyte biology could represent a novel role of DNA repair failure syndromes in obesity pathogenesis.

## Introduction

Incidence of obesity in humans has nearly tripled worldwide in the past 50 years (Wardle et al., 2008; Brandkvist et al., 2019). This exponential rise cannot be assigned to a single cause as obesity and its related traits are genetically and environmentally complex. Body weight and body mass index (BMI) are some of the most heritable traits that have been measured in humans, with as much as 80% of the trait variance attributable to genetic factors (Wardle et al., 2008; van der Klaauw and Farooqi, 2015). Variants identified in genome-wide association studies on these phenotypes account for barely 5% of observed variance (Brandkvist et al., 2019), which neatly illustrates the genetic complexity of obesity and its related traits. Genetically tractable animal models such as the rat remain a valuable tool to elucidate the contributions of common loci or rare variants on complex diseases such as obesity or any condition for which it is a risk factor.

The Lyon Hypertensive (LH) rat is a model of spontaneous moderate hypertension exacerbated by dietary salt, obesity, dyslipidemia, and insulin resistance; as such it has been used as a genetic model of MetS (Kwitek, 2019). In fact, the LH rat is the only known hypertensive rat with a higher body weight than its normotensive control (Sassolas et al., 1981). The LH rat is useful as a model of genetic discovery due to the existence of a closely related strain, the Lyon Normotensive (LN) rat, which is normotensive, metabolically healthy, and resistant to dietary or pharmacological metabolic challenges (Vincent et al., 1997; Martin-Galvez et al., 2017). The genomes of the LH and LN rat strains have been sequenced (Atanur et al., 2013), and have demonstrated close genetic relatedness and minimal genetic variance (Ma et al., 2014), which lends itself to fine-mapping of causal loci. The importance of LH chromosome 17 (chr17) to their metabolic dysfunction has been demonstrated on multiple occasions, when LH consomic strains produced with either Brown Norway (Gilibert et al., 2008) or LN (Bilusic et al., 2004; Ma et al., 2017) chr17 replacement afforded some protection against obesity, dyslipidemia, and hypertension.

Independent QTL have been identified for most features of MetS on chr17, including blood pressure, body weight, adiposity, and dyslipidemia (Bilusic et al., 2004; Wang et al., 2015; Ma et al., 2017). MetS susceptibility is polygenic in the LH rat, thus large QTL such as those generated by previous F2 intercross or backcross mapping studies may contain numerous candidate genes, necessitating additional breeding schemes (Clark and Kwitek, 2021). The creation of congenic models allows entire QTL to be substituted into a genomic background, potentially preserving important gene-gene interactions or necessary regulatory context in a way that targeted knockouts or mutations cannot (Clark and Kwitek, 2021). The purpose of this work was to fine-map the proximal end of a previously identified body weight QTL (Bw32; RGD: 1354596) (Bilusic et al., 2004) on chr17 by characterization of a novel congenic rat strain and to identify a putative candidate gene underlying the body weight differences seen in the chr17 consomic rats (Gilibert et al., 2008; Ma et al., 2017).

## Materials and Methods

### Generation of Congenic Animal Model

**FIGURE 1:**
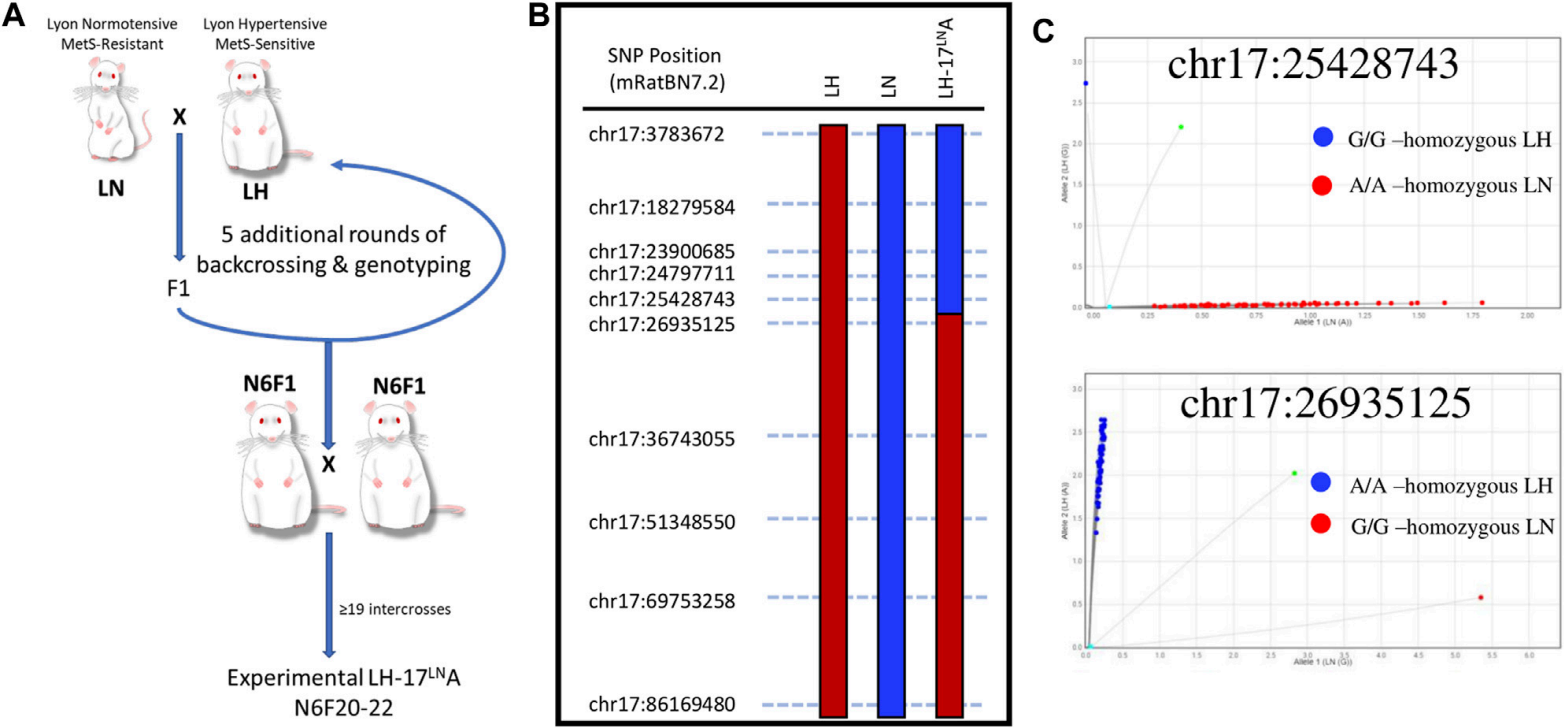
Generation and validation of a novel LH congenic rat to study the genomic basis of Metabolic Syndrome. **(A)** The LH.LN-Chr17^LN^-(Fancc^rgdv551196202-C^-rs107291522)/Aek congenic line (LH^17^LNa) was generated by crossing an LH male rat to an LN female rat, and then backcrossing F1 males to females of the LH strain. Between generations N1-N6, males were genotyped genome-wide before selection for breeding to rapidly fix the genetic background (except for chromosome 17) for LH variants. Starting at N generation 6, male and female rats were intercrossed to homozygous animals for the chr17 region of interest. This strain was maintained by intercrossing in this manner until generation N6F20, before LH^17^LNa animals were enrolled for experiments. **(B)** The congenic line LH^17^LNa is defined by their genotypes at the twelve SNPs throughout chr17, where animals are homozygous LN (blue) up to chr17:25428743 and homozygous LH (red) starting at chr17:26935125. **(C)** Fluorescent genotyping calls for the two SNP markers that define the boundary of the LH^17^LNa congenic confirm that all experimental animals used in this study were homozygous for the LN allele at position chr17:25428743 (top) and homozygous for the LH allele at position chr17:26935125 (bottom).

A congenic rat with proximal rat chromosome 17 introgressed from the LN strain to the LH strain was generated using a “speed congenic” method at the University of Iowa (Wakeland et al., 1997; Ma et al., 2017) (Figure 1). In brief, an LH (LH/MavRrrc; RRID:RRRC_00057) male was bred to an LN (LN/MavRrrc; RRID:RRRC_00058) female to generate F1s. An F1 male was subsequently backcrossed to an LH female, and progeny were genotyped with SNPs spanning chromosome 17 to select heterozygous breeders for the subsequent generations, until backcross generation 4 (N4). Between N4 to n generation 6 (N6), candidate males that retained chr17 heterozygosity were screened using a custom panel of 453 SNPs, designed to tag all major LH/LN haplotypes genome-wide, as previously described (Ma et al., 2017). Starting at N6, animals that were heterozygous for targeted regions on chr17 but homozygous for the LH allele in the background genome were brother-sister mated for several generations to inbreed the line, producing the novel congenic strain LH.LN-Chr 17^LN^-(Fancc^rgdv551196202-C^-rs107291522)/Aek (RRID:RRRC_00825; RGD:13207341; LH^17^LNa) (Figure 1A). LH^17^LNa congenic rats from the N6F20-F22 generations were used for the experiments at the Medical College of Wisconsin.

The LH/MavRrrcAek (RRID:RGD_10755352) animals were maintained simultaneously as an inbred line, first at the University of Iowa, then at the Medical College of Wisconsin, and animals from generation F26-29 were used as experimental controls for the LH^17^LNa congenic line. DNA for genotyping was isolated from tail tissue (DNeasy Blood and Tissue kit, QIAGEN) according to the manufacturer’s specifications. A panel of 12 SNPs were genotyped utilizing an allele-specific amplification method (rhAmp reagents: Genotyping Master Mix, Cat#1076446; rhAmp Reporter Mix with Reference Dye, Cat#1076450) with custom fluorescent genotyping assays (rhAmp SNP assays, IDT, Coralville, IA) that were designed to tag segregating loci along the length of chromosome 17 (Figure 1B; Supplementary Table S1). Phenotyped animals were tested using this panel to confirm that LH^17^LNa animals are homozygous for the LN genotype at all segregating loci up to chr17:25428743 and are homozygous for the LH genotype at all loci after chr17:26935125 (Figure 1C). All referenced genome positions are based on the newest rat genome assembly (mRatBN7.2) (Howe et al., 2021). Variants from the LH and LN strains are publicly available for download at: https://download.rgd.mw.edu/strain_specific_variants/Dwinell_MCW_HybridRatDiversityProgram/.

All animal breeding and phenotyping was performed in accordance with institutional policies and was approved by the Institutional Animal Care and Use Committees at the University of Iowa and the Medical College of Wisconsin. All animals were housed in micro-isolator caging on a 12–12 h light-dark cycle at the University of Iowa and a 14–10 h light-dark cycle at the Medical College of Wisconsin. Animals at the University of Iowa were provided Teklad 7913 diet, and reverse osmosis filtered water. Animals at MCW (including all experimental animals) were provided a continuous source of enrichment and maintained on a soy protein-free chow (Teklad 2920X) and hyperchlorinated reverse osmosis filtered water.

### Metabolic Phenotyping Protocol

In total, sixty-four LH rats (35 female/29 male) and sixty-three LH^17^LNa congenic rats (33 female/30 male) were used for this study. All animals were weaned at 3 weeks of age, and body weight was tracked weekly until 15 weeks of age. Body composition (fat mass, fat free mass = total body weight-fat mass) was measured at 8, 11, and 14 weeks of age using time-domain nuclear magnetic resonance (TD-NMR; LF110, Bruker Biospin). At 14 weeks of age, all animals were placed into metabolic cages (#40615, Lab Products, Inc.) for measurements of food and water intake and feces and urine output. Food and feces samples were collected for estimates of digestive and energy efficiency. In brief, feces samples were desiccated (60°C for 3–4 days), pressed into ∼200 mg pellets, and combusted to completion in a semi-micro bomb calorimeter (6725; Parr Instruments). Digestive Efficiency was calculated as the calories absorbed divided by calories ingested, and Energy Efficiency was defined as body mass change divided by calories absorbed over the final 48 h in metabolic cages as described in (Reho et al., 2022).

At 15 weeks of age, animals were fasted overnight before they were euthanized with an overdose of CO_2_. Blood was drawn through a cardiac puncture and placed into serum separator tubes (BD Microtainer Serum Separator Tubes, #365978, Becton-Dickson) and plasma separator tubes (BD Microtainer Tubes with K_2_EDTA, #365974, Becton-Dickson) for further analysis. All tissues were weighed before additional processing for RNA (RNA*later* Solution, #AM7021, Invitrogen), snap frozen in liquid nitrogen, or formalin fixed for histology.

### Multiplexed Metabolic Phenotyping and Data Analysis

A subset of the total cohort (LH: *n* = 17 females/*n* = 15 males LH^17^LN: *n* = 16 females/*n* = 10 males) were phenotyped using a multiplexed metabolic phenotyping platform (Promethion; Sable Systems International). Animals entered the Promethion housing on Monday morning and were housed continuously until Friday morning. Animals were subjected to NMR analysis immediately prior to entering and after exiting the Promethion system (Reho et al., 2022). Data presented were analyzed from the final 2 days of recording after the initial acclimation period. Heat production, meal, drink, locomotor activity, and behavior patterning were analyzed using custom macros provided by Sable Systems.

When an animal entered a period of no movement for greater than 5 min, metabolic rate during that time was averaged to obtain a “sleep metabolic rate” value. This value was normalized to the disparate fat free masses using a generalized linear model with estimated marginal means (Grobe, 2017).

### Analysis of Glucose Tolerance and Insulin Resistance

An intraperitoneal glucose tolerance test (IPGTT) was performed on a subset of the total animals used (Female LH: *n* = 11; Female LH^17^LNa: *n* = 9; Male LH: *n* = 10; Male LH^17^LNa: *n* = 10) between 10 and 12 weeks of age. Rats were fasted for 6 h between 5AM and 11AM, before a baseline blood draw and glucose check via the tail vein. All animals were then given a (1 mg/kg) intraperitoneal dextrose injection (Cat#1046864; Henry Schein), and blood was sampled at regular intervals via tail vein for glucose (Contour Next, Cat#7278, Ascensia Bayer). Plasma was collected concurrently into tubes (Sarstedt Microvette Tubes with Heparin, Cat#NC9046728; Fisher Scientific) to measure insulin, according to the manufacturer’s specifications (Rat Insulin ELISA, Cat#80-INSRT-E01, ALPCO).

### Analysis of Circulating and Urinary Biomarkers

Triglycerides were extracted from ∼100 mg of snap frozen female liver tissue in 5 ml of extraction buffer (5 volumes isopropanol: 2 volumes water: 2 volumes Triton X-100) via bead beating and centrifuged to remove supernatant. Prior to running the assay (BioAssay Triglyceride Assay Kit, Cat#ETGA-200, both plasma and liver homogenates were diluted 1:5 in water, and the assay was run as directed. Total cholesterol, and High Density Lipoprotein (HDL) and Low Density Lipoprotein (LDL)/Very Low Density Lipoprotein (VLDL)-cholesterol fractions were measured in female serum samples from the terminal blood draw using the EnzyChrom AF HDL and LDL/VLDL Assay Kit (Cat# E2HL-100, BioAssay Systems) following the manufacturer’s instructions.

Plasma samples from the terminal blood draw were used for analysis of leptin and adiponectin. Plasma leptin was measured in female LH (*n* = 29) and LH^17^LNa (*n* = 22) rats using the Rat Leptin ELISA kit (Product #: 90040; Crystal Chem). Adiponectin was measured in diluted plasma (1:6000) in LH (*n* = 17) and LH^17^LNa (*n* = 17) female rats using a Rat Adiponectin ELISA kit (Product #80570; Crystal Chem). Urinary corticosterone was measured in 24-hour urine samples collected from metabolic cages after 48 h of acclimation. For corticosterone (Cat#K014-H5; Arbor Assays), female urine samples were diluted 1:20 and run according to manufacturer’s recommendations. Samples that fell outside the standard curve were further diluted (1:40) and re-run.

All immunoassays listed above were read on a CLARIOstar plate reader (BMG Labtech). Results for all immunoassays were quantified using a 4-parameter logistic regression web tool available at MyAssays.com.

### Histological Examination of Perirenal Fat Tissues

Female perirenal white adipose tissue (PWAT) was formalin-fixed and paraffin-embedded before sectioning and H&E staining. Sections were scanned with a Hamamatsu slide scanner (Children’s Research Institute Imaging Core, Medical College of Wisconsin) and .ndpi image files were analyzed for both cell counts and average area using Nanozoomer software (Hamamatsu). For each animal, six 0.25 mm^2^ areas were quantified for average number of adipocytes within the section (LH: *n* = 14; LH^17^LNa: *n* = 16) as well as average area of adipocytes within the section (LH: *n* = 15; LH^17^LNa: *n* = 16).

### Gene Expression Analysis in Perirenal Adipose Tissue

RNA from approximately 250 mg of female PWAT was isolated via bead beating (Tubes: Cat#15-340-157; Bead Mill 4: Cat# 15-340-164; Fisherbrand) using TRIzol reagent (Cat#15596018; Ambion) and chloroform (Cat# BP1145-1; Fisher Chemical) extraction. Samples were purified using the RNeasy Mini Kit (Cat#74134; QIAGEN). RNA concentration and purity was confirmed on a Nanodrop and through agarose gel electrophoresis. 2.5 ug of RNA was reverse transcribed into cDNA using the iScript cDNA synthesis kit (Cat#1708891, BioRad), with all manufacturer’s directions followed, except incubation times were doubled. This kit uses a blend of oligo dTs and random hexamers for priming. FAM-labelled, double-quenched (ZEN, IABlk) gene expression assays (Supplementary Table S2) and reagents from the PrimeTime line (IDT, Coralville, IA, United States) were used according to manufacturer’s directions and run using the recommended fast reaction parameters, with 50 ng of cDNA per triplicate reaction. All experiments were run on a Quant Studio 3 (Applied Biosystems). Gene expression data were analyzed using the 2^−ddCt^ method, with hypoxanthine phosphoribosyltransferase 1 (*Hprt1*) used as the internal control (Livak and Schmittgen, 2001).

### LH/LN Variant Filtering and Candidate Gene Prioritization

Genomic variants from whole genome sequence were identified in LH/MavRrrcAek and LN/MavRrrcAek compared to the rat reference genome mRatBN7.2 (Howe et al., 2021) as part of the Hybrid Rat Diversity Program. Variant (vcf) and alignment (BAM) files are available for download from the Rat Genome Database: (https://download.rgd.mcw.edu/strain_specific_variants/Dwinell_MCW_HybridRatDiversityProgram/). From the vcf files, variants identified in LH/MavRrrcAek and LN/MavRrrcAek strains in the region of chromosome 17 that differs between the LH and LH^17^LNa congenic strains were then sorted by zygosity, and heterozygous variants were removed. The effects of genetic variants on genes were evaluated using SnpEff (Cingolani et al., 2012). BAM files were reviewed for all identified variants of high and moderate impact and discarded when they localized to regions where the assembly was suspect. Remaining positions were sequence verified. Sequence flanking the putative variant was PCR amplified according to the manufacturer’s specifications using 50 ng of spleen gDNA, 500 ng primers (Supplementary Table S6), and Q5 Hot-Start High-Fidelity 2X Master Mix (Cat# M0494S, New England Biolabs) before verification by Sanger sequencing (Functional Biosciences, Inc., Madison, WI). Finally, the functional impact of each amino acid substitution or indel was estimated by Provean, which was used because it can predict the effects of large amino acid deletions in any species (Choi and Chan, 2015).

### Statistical Analysis of Physiological Endpoints

Prior to statistical analysis, all data were checked for outliers using a ROUT test (GraphPad) and were checked for normal distribution using a Shapiro-Wilk test and equal variance using an F test. Analyses between two groups that passed these tests used an unpaired two-tailed t-test to compare the data. Data that were not normally distributed after outlier exclusion were compared using a Mann-Whitney nonparametric test, while data that failed the equal variance test were compared using an unpaired two-tailed t-test with Welch’s correction.

For analysis between two groups with time as an additional variable, a two-way ANOVA with repeated measures was used for complete data sets, while a mixed model was used for datasets with missing data points. Sidak’s correction was used for multiple testing.

For fat free mass adjusted metabolic rate, a generalized linear model with estimated marginal means was used as previously described (Grobe, 2017). Strain effect was considered significant if *p* value was less than 0.05 after adjusting for covariates.

All statistical tests were conducted in GraphPad Prism version 8, except for the generalized linear modeling test, which was performed in SPSS version 27. Data in tables are presented as Mean ± SEM.

## Results

### Generation and Validation of a Novel LH Congenic Rat Model to Study the Genomic Basis of Metabolic Syndrome

Utilizing F2 intercross designs, QTL and liver eQTL mapping experiments demonstrated a novel locus in the LN genome that contributed to traits such as decreased blood pressure (Bilusic et al., 2004; Wang et al., 2015) and decreased plasma leptin concentration (Wang et al., 2015). Further dissection of the mapped locus on chromosome 17 for blood pressure and adiposity-related traits revealed an overlapping cis-eQTL controlling the expression of the uncharacterized gene, *C17h6orf52* (*RGD1562963*) (Wang et al., 2015). The discovery of these QTLs associated with MetS led to the generation of the novel congenic rat strain LH^17^LNa, whereby the genomic area on chromosome 17 containing the LN QTL was introgressed on the genome of the LH strain using a speed congenic approach (Figure 1A). SNP markers along the length of chromosome 17 were selected to validate the parental genomes and genotyping was performed at each marker to fine map the boundaries of the LH^17^LNa congenic rat (Figure 1B). Experimental animals were genotyped in several positions on chromosome 17 to confirm genotype on either side of the breakpoint (Figure 1C). LH^17^LNa rats were confirmed to be homozygous for the LN genotype at chr17:25428743 (Figure 1C, top) and homozygous LH at the next marker, located at chr17:26935125 (Figure 1C, bottom).

### LH^17^LNa Rats Display Female-specific Increases in Adiposity and Altered Body Composition

**FIGURE 2:**
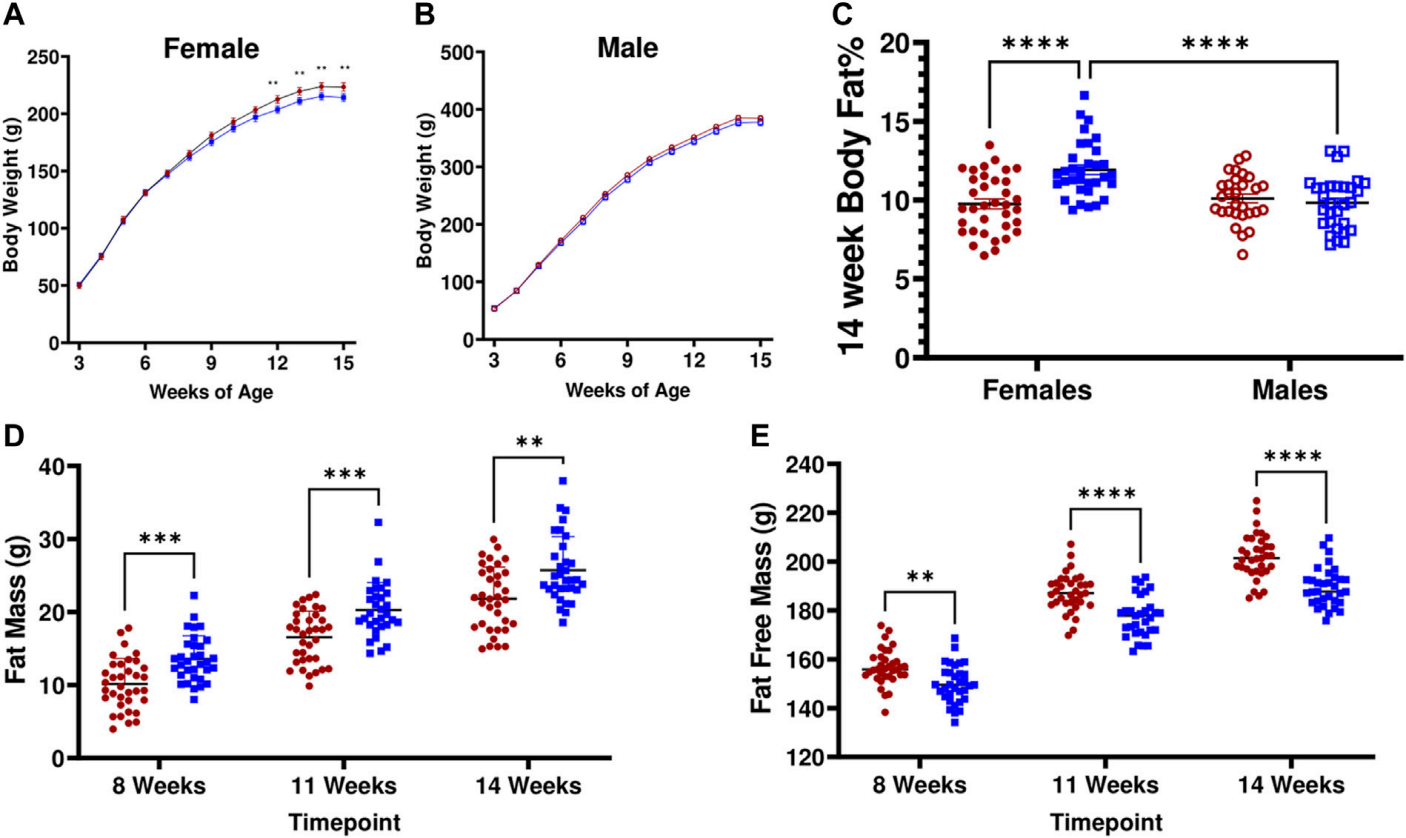
LH^17^LNa congenic rats display female-specific increases in adiposity and altered body composition. **(A)** LH^17^LNa congenic females (blue solid square, *n* = 33) and LH control females (red solid circle, *n* = 35) develop decreased body weights starting at 12 weeks of age (Two-Way ANOVA, with Sidak’s correction for multiple comparisons; strain (*p* = 0.0203) and time x strain (*p* < 0.0001)). Timepoints which were significant after Sidak’s correction are indicated by ** (*p* < 0.01). **(B)** LH^17^LNa congenic males (blue open square, *n* = 30) and LH control males (red open circle, *n* = 29) displayed no differences in body weight. **(C)** LH^17^LNa females exhibit significant sex-specific effects on body fat % (p_sex_<0.01, p_strain_<0.01, p_interaction_<0.0001), while no differences between male LH controls and LH^17^LNa congenics were noted. **(D,E).** LH^17^LNa females demonstrated increased fat mass (p_strain_ <0.0001), and decreased fat free mass (p_strain_ <0.0001, p_interaction_<0.001) at all measured timepoints (Mixed model with Sidak’s correction for repeated measures).

Consistent with the previously observed contribution of the LN QTL, LH^17^LNa females exhibit decreased weight gain (Figure 2A; *p* < 0.05 via two-way ANOVA with Sidak’s correction for repeated measures; p_growth_<0.05, p_rate_<0.05). This effect was not observed in male LH^17^LNa rats relative to LH controls (Figure 2B; Table 1). All male and female rats of both strains underwent body composition analysis via time-domain nuclear magnetic resonance (NMR), and while only minor and sporadic differences in male body composition measures were noted (Table 1), sex effect on body fat % was significant (Figure 2C, p_sex_<0.01, p_strain_<0.01, p_interaction_ <0.0001 via two-way ANOVA). Further investigation of increased adiposity of LH^17^LNa females yielded multiple observations via NMR of increased fat mass (Figure 2D; p_strain_<0.0001, Mixed model with Sidak’s correction), as well as concurrent decreases in fat free mass in the LH^17^LNa congenic females relative to control LHs (Figure 2E, p_strain_ <0.0001, p_interaction_<0.001; Mixed model with Sidak’s correction).

**TABLE 1:** Male phenotypes.

Phenotype	LH male (*n* = 29)	LH^17^LNa male (*n* = 30)	*p*-value
Body mass			
Body mass (g) at 8 weeks	252.8 ± 1.494	247.6 ± 2.805	0.1071^#^
Body mass (g) at 11 weeks	333.8 ± 1.719	327.3 ± 2.848	0.0568^#^
Body mass (g) at 14 weeks	385.2 ± 2.248	376.9 ± 3.047	0.0332*
Body composition			
Body Fat % at 8 weeks	6.600 ± 0.255	7.352 ± 0.242	0.0364*
Fat mass (g) at 8 weeks	16.80 ± 0.679	18.40 ± 0.706	0.1079*
Fat Free mass (g) at 8 weeks	237.2 ± 1.483	231.1 ± 2.825	0.0638^#^
Body Fat % at 11 weeks	8.367 ± 0.279	8.736 ± 0.260, *n* = 26	0.341*
Fat mass (g) at 11 weeks	27.96 ± 0.953	28.58 ± 0.960, *n* = 26	0.6518*
Fat Free mass (g) at 11 weeks	306.1 ± 1.733	297.9 ± 2.580, *n* = 26	0.010*
Body Fat % at 14 weeks	10.11 ± 0.277	9.821 ± 0.304	0.4934*
Fat mass (g) at 14 weeks	38.99 ± 1.085	37.12 ± 1.284	0.273*
Fat Free mass (g) at 14 weeks	346.8 ± 2.214	340.3 ± 2.599	0.0604*
Organ masses			
Left ventricle mass (g)	1.004 ± 0.010	0.964 ± 0.011	0.0111*
Adjusted left ventricle mass (mg/g)	2.610 ± 0.024	2.551 ± 0.022	0.0811*
Total kidney mass (g)	2.144 ± 0.027	2.021 ± 0.022	**0.0014** ^**$**^
Adjusted total kidney mass (mg/g)	5.572 ± 0.059	5.349 ± 0.037	**0.0021** ^**$**^
Thymus gland mass (mg)	481.7 ± 0.014, *n* = 8	442.1 ± 0.008, *n* = 11	0.0163*
Adjusted thymus gland mass (mg/g)	1.258 ± 0.031, *n* = 8	1.147 ± 0.021, *n* = 11	0.0067*
Metabolic phenotypes			
Average heat production (kcal/h)	2.299 ± 0.0242, *n* = 15	2.186 ± 0.0413, *n* = 10	**0.019** ^*^
FFM-adjusted Sleep Metabolic rate (kcal/h)	2.079 ± 0.046, *n* = 15	1.994 ± 0.057, *n* = 10	0.271 GLM (FFM = 293.9)
Total meters/48 h	110.0 ± 21.65, *n* = 12	232.2 ± 61.33, *n* = 8	0.0938^#^
Food intake (g)/48 h	41.83 ± 0.712, *n* = 15	38.19 ± 0.525, *n* = 10	0.0011*
Feeding event counts/48hr	89.93 ± 3.208, *n* = 15	90.70 ± 3.229, *n* = 10	0.8729*
Meal size (g/feeding event)	0.531 ± 0.0197, *n* = 15	0.477 ± 0.0178, *n* = 10	0.0545^$^
Feeding event duration (min)	3.029 ± 0.081, *n* = 15	3.113 ± 0.158, *n* = 10	0.6088*
Meal eating speed (food g/min)	0.109 ± 0.005, *n* = 15	0.108 ± 0.009, *n* = 10	0.683^$^
Inter-meal interval (min)	34.52 ± 1.44, *n* = 15	33.37 ± 1.08, *n* = 10	0.566*

*via Unpaired two-tailed t-test; ^#^via Welch’s unpaired two-tailed t-test; ^$^via Mann-Whitney, significant values are bolded.

The masses of several other non-adipose tissues were also decreased in the LH^17^LNa female rats: liver, left ventricle of the heart, total kidney, and thymus (Supplementary Table S3; *p* < 0.05; unpaired, two-tailed t-test). Male LH^17^LNa rats had no alterations at adipose tissue beds (Supplementary Table S4), however, decreases in both left ventricle (Table 1; *p* < 0.05, unpaired two-tailed t-test) and total kidney mass were observed (Table 1; *p* < 0.01; Mann-Whitney).

To further evaluate their metabolic health, female LH^17^LNa congenic rats and LH rat controls were phenotyped for additional MetS traits, including measures of circulating lipids and glucose tolerance and insulin resistance tests. No differences were seen in female plasma and liver triglycerides, female serum total cholesterol, HDL/LDL cholesterol ratio, nor in IPGTT responses in the LH^17^LNa females relative to the LH control females (Supplementary Figures S1A–F), suggesting this congenic rat models certain but not all components of MetS that were previously mapped to chr17. Circulating triglycerides were measured in male rats (LH: *n* = 25; LH^17^LNa: *n* = 18) but no differences were noted (data not shown). Blood was collected from male rats during IPGTT for glucose measurements, but no differences were observed (data not shown).

### Metabolic Phenotyping Revealed Differences in Energy Expenditure in Female LH^17^LNa

**FIGURE 3:**
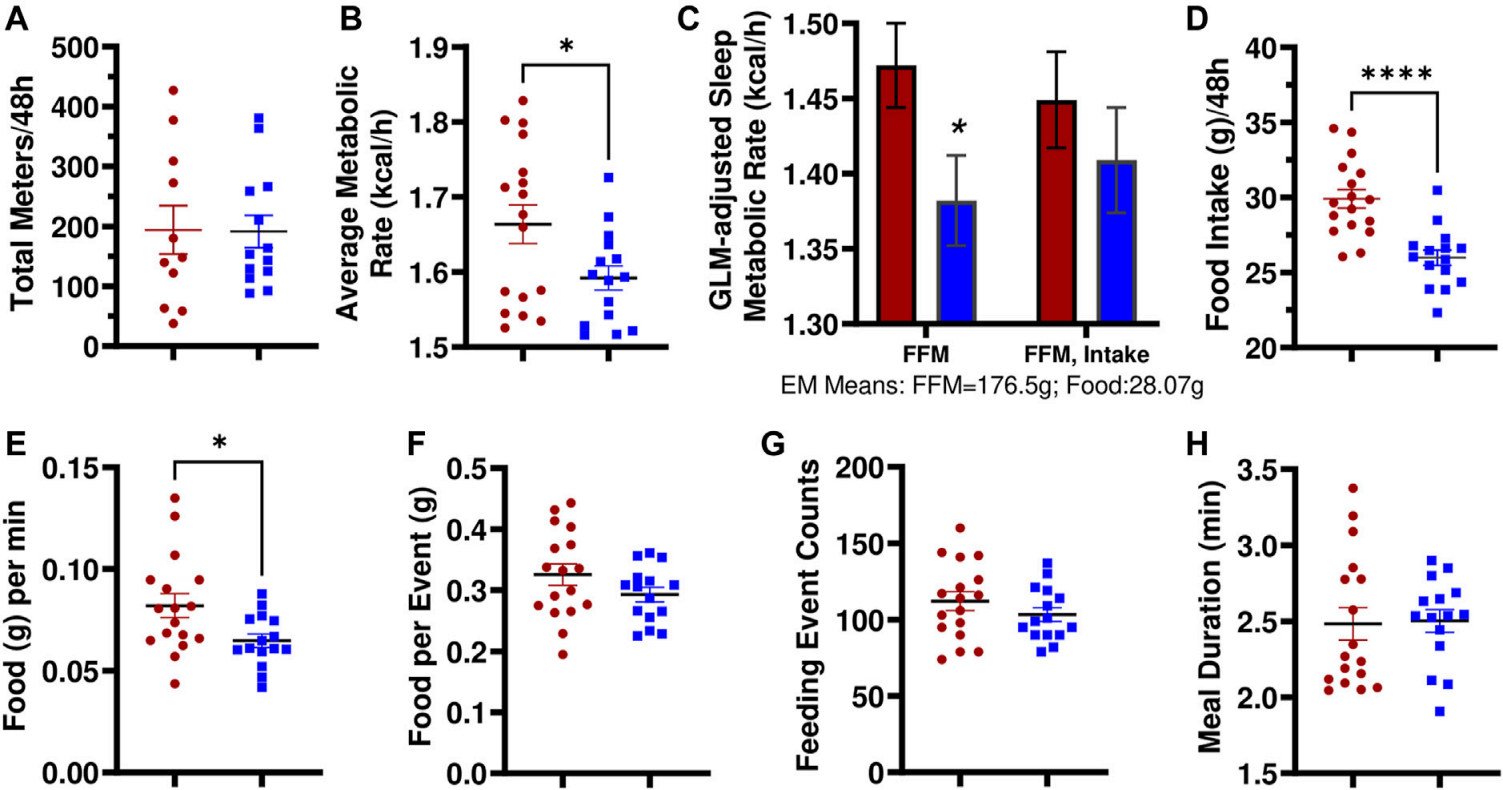
LH^17^LNa females exhibit significant decreases in metabolic rate and food intake. **(A)** LH and LH^17^LNa females do not differ in their total levels of activity (meters traveled) (LH: red circle, *n* = 11; LH^17^LNa: blue square, *n* = 13; *p* = 0.955, unpaired two-tailed t-test). **(B)** LH^17^LNa females’ average metabolic rate is lower (*p* = 0.03, unpaired two-tailed t-test) compared to control LH females during the 48-hour data collection period. **(C)** Average metabolic rate during rest (defined as a period longer than 5 min of no activity) was decreased in the LH^17^LNa strain (blue bars) (*p* = 0.035) after correction for the animals’ disparate fat free masses, but not once food intake was included as a covariate (Generalized Linear Model with Estimated Marginal Means: FFM:176.5g, Food Intake: 28.07g). **(D)** LH^17^LNa females ingest less food during the Promethion measurement period (*p* < 0.0001, unpaired two-tailed t test). The decreased food intake is driven by **(E)** reduced eating speed (*p* = 0.0128, unpaired two-tailed t-test). No differences were seen in **(F)** total food intake per meal (*p* = 0.143, unpaired two-tailed t-test), **(G)** total number of meals (*p* = 0.273, unpaired two-tailed t-test), or **(H)** meal duration (*p* = 0.682, Mann Whitney). Unless otherwise specified, LH female: red circle, *n* = 17, and LH^17^LNa female: blue square, *n* = 15.

Utilizing multiplexed metabolic phenotyping (Promethion), the LH^17^LNa congenic strain was found to exhibit differences from LH in energy expenditure. LH^17^LNa females did not differ in measures of activity (Figure 3A; Supplementary Figure S2A), or in sleep/wake patterns (Supplementary Figure S2B). The average metabolic rates of the LH^17^LNa females were decreased (Figure 3B; *p* < 0.05; unpaired two-tailed t-test), and this difference remained after correction for their decreased fat free masses (Figure 3C, *p* < 0.05 *via* general linear modeling with estimated marginal means, covariate FFM = 176.5g). Average metabolic rate was decreased in male LH^17^LNa congenics as well (Table 1; *p* < 0.05, *via* unpaired two-tailed t-test). Additionally, total food intake in LH^17^LNa females was decreased compared to LH female controls (Figure 3D; *p* < 0.0001; unpaired two-tailed t-test), and this difference accounts for the strain differences in metabolic rate that were observed (Figure 3C, *p* = 0.468 via general linear modeling with estimated marginal means, covariate: FFM = 176.5g, Food Intake = 28.07g). During each individual feeding event, the LH^17^LNa females ate fewer grams per minute (Figure 3E; *p* < 0.05; unpaired two-tailed t-test), despite no differences in total food per meal (Figure 3F), total number of meals (Figure 3G), meal duration (Figure 3H), or inter-meal interval (Supplementary Figure 2C). The significant reduction in food intake seen in the LH^17^LNa females was also corroborated in metabolic cages at 14 weeks of age (Supplementary Figures S2D,E). Feces collected from metabolic cages was dried and combusted to estimate animals’ digestive efficiency (Supplementary Figure S2F) and energy efficiency (Supplementary Figure S2G) over the period the animals were in metabolic caging. No changes to either digestive efficiency (Supplementary Figure S2F), or energy efficiency (Supplementary Figure S2G) were observed in females. Corticosterone was measured in a 24-hour urine sample to test for stress-induced anorexia while in the metabolic caging, however there were no changes to the levels of urinary corticosterone in the LH^17^LNa females relative to LH control females (Supplementary Figure S2H).

Male LH^17^LNa rats also ate less total food while housed in the Promethion (Table 1) and in metabolic cages (Supplementary Table S4) relative to male control LHs—one of the few phenotypes present in both sexes. Additionally, male LH^17^LNa rats had a decreased overall metabolic rate (Table 1), but this was not significant after accounting for fat free mass with a generalized linear model (Table 1). In contrast to female LH^17^LNa rats, food intake per meal trended down in male LH^17^LNa rats (Table 1; *p* = 0.0545; Mann-Whitney), but eating speed was unaffected (Table 1). In male LH^17^LNa rats, number of meals and meal timing was unaffected (Table 1).

### Increased Adiposity in LH^17^LNa Rats Is Associated With Differences in Fat Distribution and Hypertrophic Abdominal Adipocytes

**FIGURE 4:**
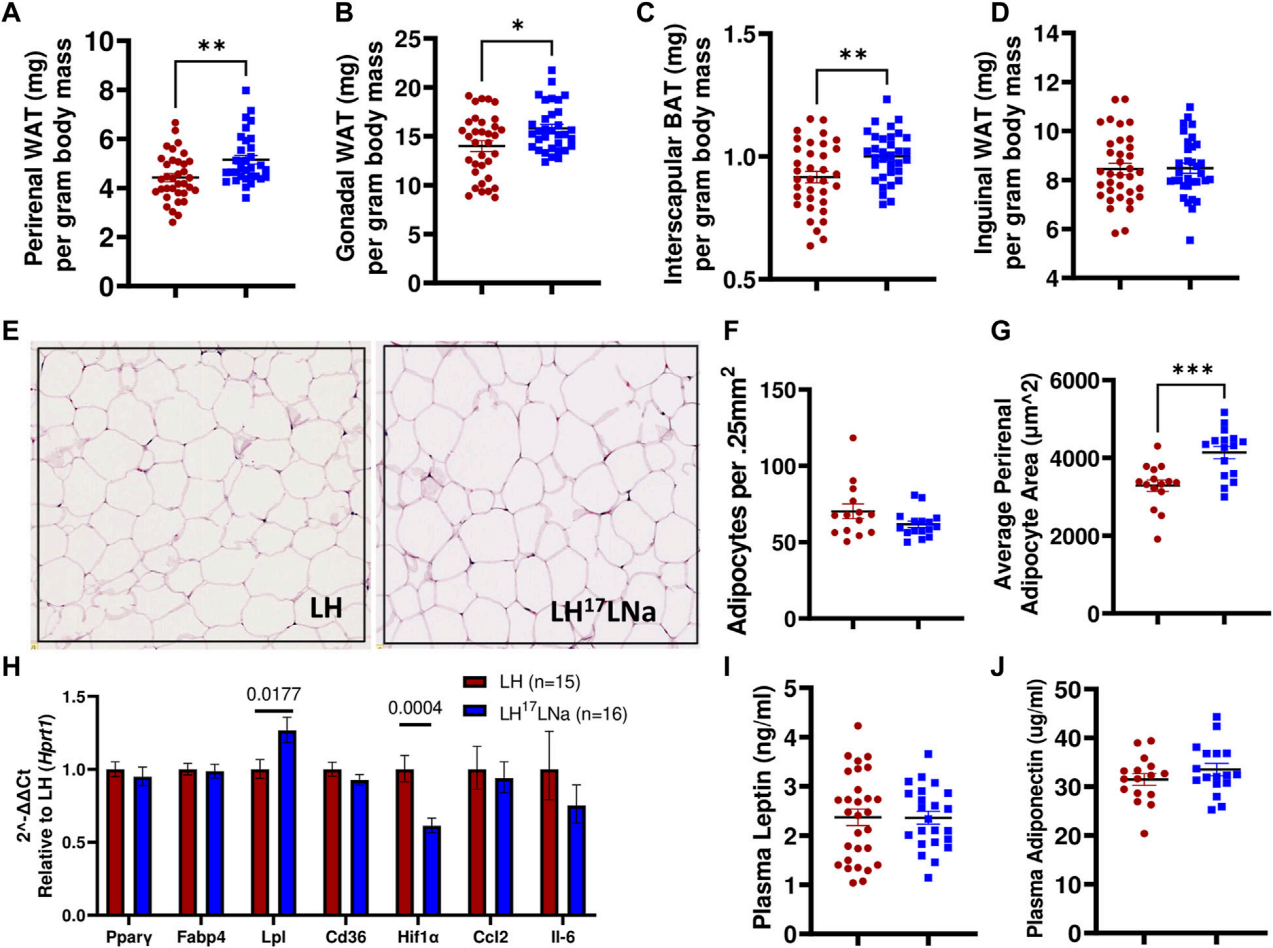
Adiposity differences are specific to abdominal white and interscapular brown adipose depot masses. **(A)** Perirenal white adipose mass (*p* < 0.001, Mann-Whitney) and **(B)** gonadal white adipose mass was increased in LH^17^LNa females compared to LH controls (LH: *n* = 34; LH^17^LNa: *n* = 33; *p* < 0.05, Mann-Whitney). **(C)** Relative interscapular brown adipose mass was increased in LH^17^LNa females compared to LH controls (*p* = 0.006, unpaired two-tailed t-test). **(D)** No changes were noted in inguinal adipose tissue mass. **(E)** Representative perirenal tissue sections stained with H&E from the LH and LH^17^LNa strains are shown. Perirenal tissue sections were quantified for both **(F)** average cell number (LH: *n* = 14; LH^17^LNa: *n* = 16) and **(G)** average cell size (LH: *n* = 15; LH^17^LNa: *n* = 16, *p* < 0.001, unpaired two-tailed t-test). **(H)** Rt-qPCR on female PWAT RNA (LH: *n* = 15, LH^17^LNa: *n* = 16) was done to assess expression of *Pparγ* and several of its target genes (*Fabp4*, *Lpl*, *Cd36*), as well as fibrosis marker *Hif1α* and immune markers *Ccl2* and *Il6*. *Lpl* (lipoprotein lipase) was significantly upregulated (*p* = 0.0177, unpaired two-tailed t-test) in this tissue, expression of fibrosis marker *Hif1α* was decreased (*p* = 0.0004, unpaired two-tailed t-test); all other tested genes did not differ. Circulating adipokines **(I)** leptin and **(J)** adiponectin were not different between the LH (Leptin LH: *n* = 29; Adiponectin LH: *n* = 16) and LH^17^LNa (Leptin LH^17^LNa: *n* = 23; Adiponectin LH^17^LNa: *n* = 18) strains. Unless otherwise noted, LH *n* = 35; LH^17^LNa *n* = 33.

Consistent with the observations of body fat mass increases in the LH^17^LNa female congenic rat, abdominal (perirenal and gonadal) adipose tissue masses are increased (*p* < 0.05; Mann-Whitney), as well as interscapular brown fat (*p* < 0.01; unpaired two-tailed t-test) (Figures 4A–C), with no differences noted in the inguinal adipose fat depot (Figure 4D). Liver mass was decreased in LH^17^LNa compared to LH control before and after normalization to body mass (Supplementary Table S3) and there was no evidence of increased triglyceride deposition in the liver (Supplementary Figure S1D). We next sought to determine if the increase in total perirenal adipose mass was due to cellular hypertrophy, hyperplasia, or both. In tissue sections stained with H&E (representative images from each strain shown in Figure 4E), we found no difference in the average number of adipocytes (Figure 4F), but average adipocyte area was increased in LH^17^LNa congenic females (Figure 4G, *p* < 0.001; unpaired two-tailed t-test) suggesting that increased adiposity in LH^17^LNa females is caused by increased adipocyte area that is fat depot specific.

To determine relevant pathways underlying this phenotype, a panel of genes were selected to investigate several genes that are commonly dysregulated during adipocyte hypertrophy via RT-qPCR (Gonzales and Orlando, 2007; Cai et al., 2012; Moreno-Indias and Tinahones, 2015; Ye et al., 2022). *Lpl* (lipoprotein lipase) expression was increased in female LH^17^LNa PWAT tissue relative to the LH control (Figure 4H; *p* < 0.05; unpaired two-tailed t-test), with no changes to *Pparγ* expression, nor its target genes, *Fabp4* and *Cd36* (Figure 4H). Immune cell infiltration markers *Ccl2* and *Il6* were also not found to be differentially expressed (Figure 4H). Interestingly, *Hif1α*, commonly used as a marker of fibrosis in fat (Halberg et al., 2009) was decreased in the LH^17^LNa females relative to control LHs (Figure 4H, *p* < 0.001 via unpaired two-tailed t-test).

Plasma from 15 week old females was analyzed for leptin (Figure 4I) and adiponectin (Figure 4J), to determine whether the differences in food intake was correlated to adipokines with established connections to feeding behaviors and fat mass (Bluher, 2012). No differences were observed in either circulating levels of leptin (Figure 4I) or adiponectin (Figure 4J).

### Resequencing of the Lyon Strains Uncovers Several Potential Candidate Genes

**FIGURE 5:**
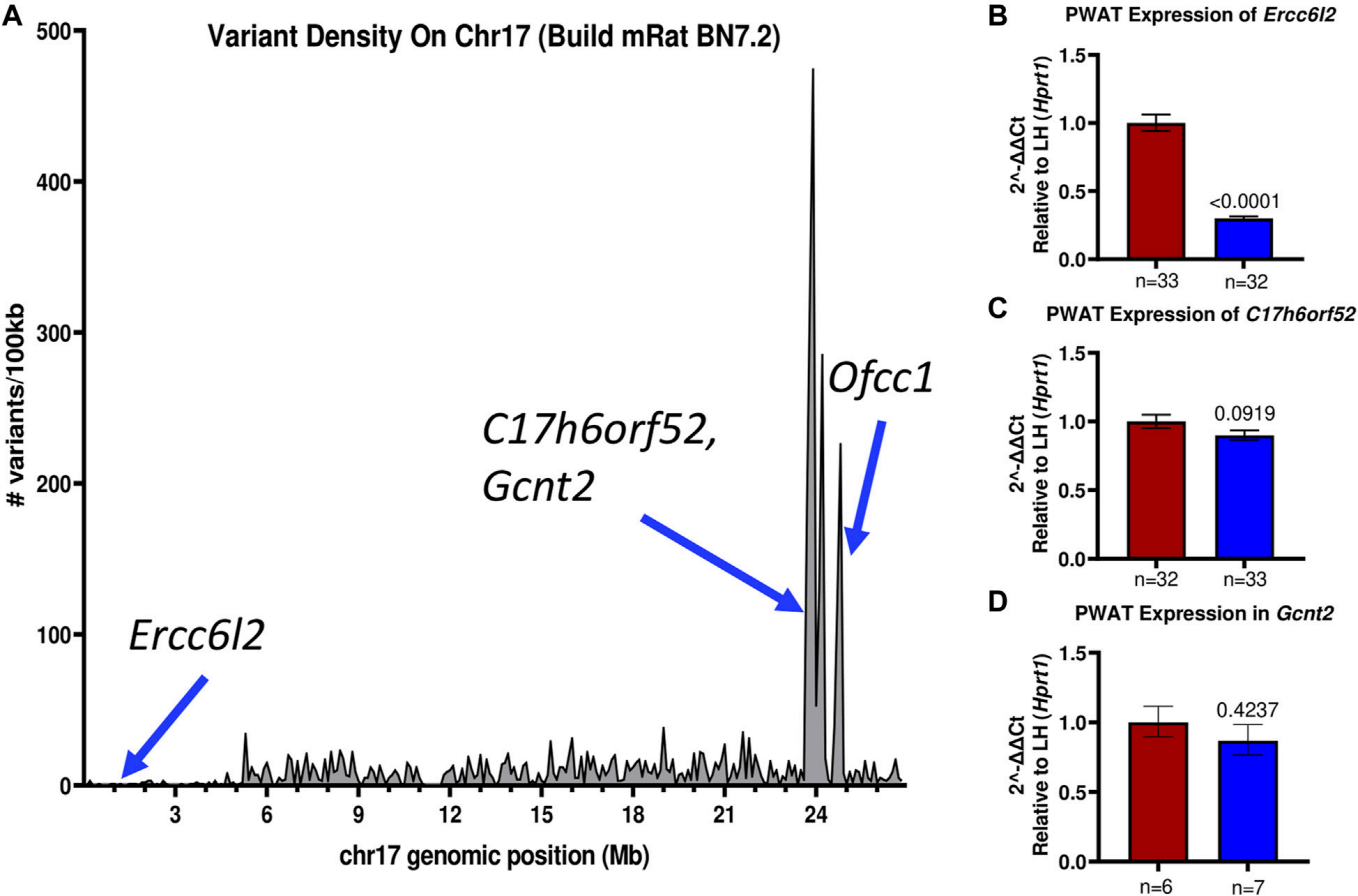
The LH^17^LNa congenic interval contains several possible candidate genes. **(A)** The LH and LN rat genomes were sequenced and aligned to mRatBN7.2. Total genomic variants unique to either the LH or LN strains were divided into 100 kilobase (100 kb) intervals and plotted to visualize the major LH/LN haplotypes. **(B–D)** Expression of potential candidate genes **(B)** *Ercc6l2*, **(C)** *C17h6orf52,* and **(D)** *Gcnt2* were analyzed in female PWAT tissues, using *Hprt1* as an endogenous control. **(B)** *Ercc6l2* is significantly downregulated (∼70%) in LH^17^LNa congenic females relative to the control LHs (LH: *n* = 33; LH17LNa: *n* = 32; *p* < 0.0001; unpaired two-tailed t-test). **(C)** *C17h6orf52* was not differentially expressed in PWAT tissue, nor was **(D)** *Gcnt2*.

Recently, the genomes of the LH/MavRrrcAek and LN/MavRrrcAek strains were sequenced and aligned to the most recent rat genome assembly, mRatBN7.2 (Howe et al., 2021), through the Hybrid Rat Diversity Program. Strain variant data are freely available from the Rat Genome Database (Tutaj et al., 2019; Smith et al., 2020). A variant density plot (Figure 5A), containing a total of 3800 homozygous SNPs and indels unique to either the LH or LN parental strains was generated through this reanalysis within the introgressed region on chr17 that differs between the LH parental and LH^17^LNa strains. SnpEff was used to assess the potential functional impact of each identified variant and assigns different variant classifications, where “HIGH” indicates a disruptive impact, “MODERATE” indicates a non-disruptive but possibly functional alteration in protein effectiveness, “LOW” indicates a mostly harmless variant, and “MODIFIER” indicates a non-coding variant, or a variant without evidence of impact (Cingolani et al., 2012). To prioritize candidate genes from this list of variants, SnpEff was used on the 1367 protein-coding variants (Supplementary Table S5). Of these, 1361 were annotated as “MODIFIERS” or “LOW” impact and were not considered further. The remaining 6 positions were grouped based on annotation of “HIGH”, or “MODERATE” impact (Table 2), and sequence verified to confirm the presence of the variant in the parent strains (Supplementary Table S6).

**TABLE 2:** Genes and variant positions within coding exons.

Annotation impact	Gene symbol	Gene name	Variant position (mRatBN7.2)	Amino acid change	Provean prediction	Variant strain
High	*Ercc6l2*	ERCC excision repair 6 like 2	chr17:1282819	p.Cys419fs	DELETERIOUS	LN
					−15.98	
Moderate	*C17h6orf52*	Similar to human chromosome 5 open reading frame 52			NEUTRAL	LH
			chr17:23762853	p.Val103Ile	−0.055	
			chr17:23771379	p.Arg202His	−0.040	
			chr17:23771391	p.Cys206Tyr	−0.572	
	*Gcnt2*	glucosaminyl (N-acetyl) transferase 2 (I blood group)	chr17:23900685	p.Ala131Thr	NEUTRAL	LN
					1.544	
	*Ofcc1*	Orofacial cleft candidate 1	chr17:24797711	pThr845Ile	DELETERIOUS	LN
					−3.767	

Note: PROVEAN scores < −2.5 were considered deleterious by the prediction tool.

Of note, the LN strain has a 5 nucleotide exonic deletion in the gene *Ercc6l2*. The variant is not located in a block of linkage disequilibrium differing between the LN and LH strains, and the nearest other variants are more than 100 kilobases away. This isolated frameshift variant results in a premature stop codon and immediate termination of the protein at amino acid 419. Further genotyping of the experimental LH^17^LNa rats confirmed that the congenic strain contains the LN allele, that is, they are homozygous for the null allele of *Ercc6l2*. Because the deletion occurs relatively early in the protein coding sequence, RT-qPCR was performed to assess the expression of *Ercc6l2* in perirenal adipose tissue. Compared to control LH females, LH^17^LNa congenic female rats express 70% less *Ercc6l2* transcript (Figure 5B; *p* < 0.05 *via* unpaired t-test), suggesting that the LN allele of this gene undergoes nonsense-mediated decay.

The remaining identified loci all reside in a major haplotype differing between LH and LN strains (Figure 5A), where the overall level of variation is high (Ma et al., 2014). Over 100 variants mapped to each of these genes, with 5′UTR variants and upstream gene variant annotations in *C17h6orf52* and *Gcnt2* that might influence gene expression, in addition to the nonsynonymous variants found. The second gene, *C17h6orf52* (Wang et al., 2015), contains 3 missense amino acid variants in the LH strain compared to the reference allele in the LN genome, although these are all predicted to be neutral by Provean (Table 2). Furthermore, in female perirenal adipose fat depots, *C17h6orf52* is not differentially expressed between the congenic strain and the LH control strain (Figure 5C), despite almost 30 sequence variants within 5 kb of the transcription start site.

A third gene, *Gcnt2* has 30 upstream and 5′UTR variants, and over 300 total variants that mapped to the annotated gene body. Despite containing the most variants of any gene in the haplotype block, and in the congenic region in general, *Gcnt2* is not differentially expressed in PWAT tissue (Figure 5D). The final gene *Ofcc1,* also contains a missense, deleterious variant in the LN genome (Table 2), but most of the additional variants are within introns and may or may not influence *Ofcc1’*s expression. In addition, *Ofcc1* is not expressed in female PWAT tissue (confirmed by qPCR, data not shown). Furthermore, the expression pattern appears to be exclusive to testes in adult rats, thus any putative differences in expression could not be tested in this female-focused study.

## Discussion

In this study, a novel LH congenic rat strain was generated to fine-map previously identified MetS related QTL on the proximal arm of chromosome 17 in the LH rat (Bilusic et al., 2004; Sassard et al., 2007; Wang et al., 2015). In the LH^17^LNa rats, several phenotypes were observed which may be related to obesity-associated traits of MetS, such as decreased metabolic rates (Figure 3C), increased abdominal adiposity (Figures 4A,B), and adipocyte hypertrophy (Figure 4G). The LH^17^LNa females also eat less in total (Figure 3D), at each meal (Figure 3F), and weigh less overall than their LH controls (Figure 2A), traits that appear to originate from the LN parental genome. The LH^17^LNa females also exhibited no differences in other key MetS phenotypes that are segregating in the LH rat, namely high triglycerides (Supplementary Figures S1C,D), HDL/LDL cholesterol ratio (Supplementary Figure S1F), or glucose tolerance (Supplementary Figures S1A,B). This data suggests that LN alleles contained within the proximal arm of chr17 underlie obesity-associated traits specifically. Regulation of blood pressure by this locus is possible and warrants future experiments, although it was beyond the scope of the current study.

Crosses between the LH and LN strains have identified Quantitative Trait Loci (QTL) contributing to features of MetS on several chromosomes (Bilusic et al., 2004; Sassard et al., 2007; Wang et al., 2015), and their degree of genetic similarity lends itself to fine-mapping of causal loci. Previous studies (Bilusic et al., 2008; Gilibert et al., 2009) determined that breeding risk loci onto resistant genetic backgrounds is usually unable to modify traits of interest, thus a reciprocal congenic (i.e., the LH allele of the QTL bred onto LN genetic background) was not studied.

F2 intercrosses have been used several times to map blood pressure and adiposity QTL to the proximal end of chr17 but have shown mixed results for body weight QTL (Bilusic et al., 2004; Wang et al., 2015). In the most recent reanalysis of the LH and LN genomes, 1931 of the total 3800 identified positions map to within 1 Mb of the eQTL identified in Wang et al., 2015 and denote a major LH/LN haplotype. Of the sixteen genes containing variants in that haplotype, *C17h6orf52*, *Gcnt2*, and *Ofcc1* are the three that have nonsynonymous amino acid changes (Table 2). Approximately 23 Mb upstream of the major LH^17^LNa haplotype is a 5 base pair deletion in *Ercc6l2* at chr17:1282819 which has not been previously reported for the LN strain (Table 2).

Glucosaminyl (N-acetyl) transferase 2 is encoded by the gene *Gcnt2*, which is the carbohydrate branching enzyme responsible for the conversion of the linear fetal i antigen to the branched I antigen that is present in the majority of adults on erythrocytes, mucosal epithelia and the eye and olfactory bulb (Bierhuizen et al., 1993; Inaba et al., 2003; Dimitroff, 2019). The p.Ala131Thr missense mutation found in Gcnt2 was predicted to have a neutral effect (Table 2) in two of its three annotated transcripts, while the exon that contains this mutation is not included in the third isoform. Annotations for Gcnt2 dysfunction mainly involve congenital cataracts (Yu et al., 2003). More recently, Gcnt2’s involvement in cell surface carbohydrate conversion has been investigated as a prognostic biomarker in various cancer types (Dimitroff, 2019), where *Gcnt2* expression is closely tied to cancer invasiveness (Mikami et al., 2016; Peng et al., 2019). Despite over 300 variants between the LH and LN strains localized within the annotated gene body of *Gcnt2*, it appears that none of these variants were sufficient to change the expression of *Gcnt2* in PWAT (Figure 5D). Taken together, a single missense allele in *Gcnt2* with a predicted neutral consequence, and no evidence of phenotypes associated with Gcnt2 mutations or expression dysregulation led to its’ removal as a plausible candidate underlying the phenotypes in the LH^17^LNa female rats.

Orofacial cleft candidate 1 (*Ofcc1*) had the second-highest number of variants and is another gene that resides in the major LH/LN haplotype between 23.7–24.7 Mb on chromosome 17 (Figure 5A). As with *Gcnt2*, the LN parental genome is the source of most of the variation within *Ofcc1*, including its only predicted exonic mutation at chr17:24797711, resulting in a missense mutation at threonine 845 that was predicted to be deleterious (Table 2). Although *Ofcc1* is expressed in the developing face of mouse embryos (Mertes et al., 2009), attempts to recapitulate human disease associations in a *Ofcc1* ^*−/−*^ mouse found no evidence of abnormal head or eye development (Ohnishi et al., 2011), and did not appear to be expressed in adults except for in testes (data not shown). Although *Ofcc1* contains numerous variants and a missense mutation that is predicted to be pathogenic in the LH^17^LNa rat, no facial abnormalities were seen in the animals, thus *Ofcc1* is not likely as a candidate gene.

The final gene with missense mutations in the major LH/LN haplotype is *C17h6orf52*, an uncharacterized protein-coding gene orthologous to *C6orf52*. This gene is conserved across most mammalian species, with the curious exception of *Mus musculus*. When the LH and LN genomes were compared, C17h6orf52 had three nonsynonymous variants, which was the most of any of the 4 prospective genes, as well as a substantial number of upstream variants close to the TSS that could potentially affect transcription factor binding. Using an F2 intercross mapping study design, Wang et al. identified an eQTL hotspot within the major haplotype that overlapped previously identified metabolic pQTL (Bw32, Spl8, Scl68, Stl13, Insul1) on chr17 (Bilusic et al., 2004) and found the *cis*-regulated gene *C17h6orf52* as the most likely driver of the eQTL (Wang et al., 2015). As a top candidate identified in that study, expression changes were confirmed via qPCR and they found an allele-specific effect on expression, where male rats homozygous for the LN allele of the marker SNP ENSRNOSNP962219 had significantly reduced liver expression of *C17h6orf52* when compared to F2 males that were homozygous for the LH allele (Wang et al., 2015). This same effect was not seen when *C17h6orf52* expression was compared in PWAT tissue of female LH and LH^17^LNa rats (Figure 5C). The LH^17^LNa congenic rats contain the reference alleles of *C17h6orf52* that are present in the LN strain (Table 2). Although the three missense mutations are annotated as benign by Provean, it is impossible to truly predict whether the LH or LH^17^LNa allele has the correct structure and function, since *C17h6orf52* is a completely uncharacterized gene, with no annotated structure, domains, or function.

*Ercc6l2* is the only gene with a high effect variant in the LH^17^LNa rats, with a Provean score of −15.98, indicating this allele is highly deleterious. Although *Gcnt2*, *C17h6orf52*, and *Ercc6l2* are widely expressed in the rat, in perirenal adipose tissue, *Ercc6l2* was the only gene differently expressed compared to the control LHs (Figures 5B–D). Taken together, the newly identified mutation in *Ercc6l2* emerges as the best candidate variant underlying the obesity and metabolic phenotypes in the LH^17^LNa strain.

Wildtype ERCC6L2 is involved in transcription-coupled nucleotide excision repair (TC-NER), DNA recombination, DNA translocation, and chromatin unwinding (Tummala et al., 2014). It is a part of the Snf2-family of helicases, a family which is responsible for remodeling chromatin to allow access to DNA repair machinery in the event of DNA damage, and then reestablishing the proper chromatin structure after the repair is completed (Ryan and Owen-Hughes, 2011). These helicase-like proteins contain 2 functional domains: an N-terminal ATP-helicase DEAD/DEAH domain, and a catalytic C-terminal helicase. Previous reports have shown that *Ercc6l2* mutations similar to what was found in the LH^17^LNa rats led to DNA damage hypersensitivity, especially DNA lesions that activate the TC-NER pathway (Tummala et al., 2014; Tummala et al., 2018). If the truncated Ercc6l2 protein can be translated in the congenic LH^17^LNa rats, the position of the mutation would result in a protein that lacks the C-terminal helicase (Supplementary Figure S3). Unfortunately, a specific rat antibody does not currently exist to determine this.

The LN allele of *Ercc6l2* (c.1256_1260delGTGGT, *p.Cys419*; RGD VID:148240080) is a frameshift that introduces a stop codon at the site of the deletion and shows clear evidence of nonsense-mediated decay (Figure 5B), and this mutation is unique amongst all sequenced rat strains, indicating that it likely arose *de novo* in the LN strain. Similar homozygous frameshift mutations in the human ortholog *ERCC6l2* have been described in autosomal recessive Bone Marrow Failure Syndrome (Jarviaho et al., 2018), which shares 86% identity with the rat gene described here. For most human cases, the main phenotypes that are reported are bone marrow hypocellularity and low blood counts, with minimal annotations for height and weight (Zhang et al., 2016; Shabanova et al., 2018). Reduced thymic cell count in a mouse *Ercc6l2* knockout has been previously described (Liu et al., 2020), and the thymus gland of male (Table 1) and female (Supplementary Table S3) LH^17^LNa animals were observed to be 8%–10% smaller than same-sex control LHs.

DNA repair mediated by the TC-NER pathway is especially important in terminally differentiated, post-mitotic cells, where repair of actively transcribed sequences is more critical for cell survival than non-transcribed sequences (Banerjee et al., 2011; Lans et al., 2019). In the LH^17^LNa females (and males to a lesser extent), tissue mass of organs like liver, heart, and thymus were significantly reduced (Supplementary Table S3), which would not only partially account for the LH^17^LNa female’s reduced fat free mass (Figure 2E), but also their reduced overall body weight (Figure 2A). The decreased overall body weight and fat free mass observed in the LH^17^LNa strain appeared to be driven rather by the failure to attain the same fat free mass as the control LH strain between 8 and 14 weeks of age as the female LH^17^LNa and LH controls approached their final adult weight (Figure 2E), and this is likely a consequence of their reduced food intake (Figure 3D).

*ERCC6L2* has 30% identity to *ERCC6* at the protein level, the causal gene for Cockayne Syndrome B (CSB). Cockayne Syndrome (CS) has three subtypes: caused by mutations in either *ERCC6* (CSB), *ERCC8* (CSA), and an as-yet unknown third gene. It is a terminal disease caused by severe and persistent transcription stress in cells due to lack of functional TC-NER and the inability to bypass transcription blocking lesions (Lans et al., 2019; Nakazawa et al., 2020), affecting virtually all body systems. In mouse models of CS and in other knockouts of *Ercc* gene family members, reduction of food intake dramatically increased lifespan and delayed accelerated aging features (Brace et al., 2013; Vermeij et al., 2016). It may be that by lowering the calorie intake and thus metabolic rate of *Ercc6* ^*−/−*^ mice, endogenous DNA damage was reduced, and transcription stress was easier to manage in those knockout models. In the LH^17^LNa strain, it is possible that their spontaneous reduction of food intake (Figure 3D) and metabolic rate (Figure 3B) act to compensate for deficiencies in Ercc6l2 function and reduces the burden placed on the transcriptome.

We recognize the following limitations in the work. Although inbred congenic models are useful for identification and fine-mapping of candidate genes these models are difficult to leverage for further mechanistic inquiry once candidate genes or variants are identified. The identified variants in other genes and intergenic spaces could possibly contribute to or influence the phenotypes presented, which would require generation of a targeted rat *Ercc6l2* knockout to fully grasp. In our filtering and candidate gene selection process, we excluded all variants that were intergenic, non-coding, or those were SnpEff had designated “LOW” or “MODIFIER” (Supplementary Table S5). We recognize the possibility that identified variants in these regions could have regulatory functions that were not considered. We also did not prioritize variants that originated from the LH genome, (i.e., LH^17^LNa rats had the reference allele). It is likely that some LH alleles (e.g., *C17h6orf52*) may be protective in an LH genetic background, and even though they are “normalized” in the LH^17^LNa congenic rat, the loss of these may be deleterious and could contribute to the observed phenotypes.

Furthermore, we did not account for estrus cycle variability in the female experimental animals, which likely contributes to variability in the dataset, and reproductive hormone differences may underlie some of the sex-specific effects we observed, as well as the relative lack of significant phenotypes seen in the LH^17^LNa males. Although we examined all the metabolic components of MetS in our model, we did not assess blood pressure or vascular function in the congenic rats.

To address these limitations, future studies are warranted, such as the development of *Ercc6l2* knockout rats to investigate the role of Ercc6l2 dysfunction in a more mechanistic way, as well as eliminating additional background variants. Sex-specific effects on many of the metabolic phenotypes could be investigated further in ovariectomized females. Reduction in food intake was one of the most notable features of the LH^17^LNa strain, and significant reductions in the food intake of LH^17^LNa rats was observed in both sexes, and in multiple phenotyping platforms. Because the reduced metabolic rate in female LH^17^LNa rats appeared to be driven by their reduction in food intake, subsequent pair-feeding experiments are required to test that hypothesis.

While the female congenic rats had an increased percentage of fat tissue relative to total body mass, the female LH^17^LNa rats weighed less than control LHs, and this was due to decreased gain in fat free mass throughout the study period. The seemingly paradoxical adiposity increase and decreased food intake that was observed in female rats may be due to sarcopenic body composition changes that differ in men versus women, driven in part by testosterone levels (Roh and Choi, 2020). It appeared that decreased food intake in both sexes of LH^17^LNa rats significantly reduced the fat free mass that female congenics attained, but the relatively higher concentrations of male hormones such as testosterone may have protected the male rats from some of these effects since they were fairly young during the study period. If true, castration or aging of the male rats may uncover a similar phenotype to what was observed in female rats. However, assessments of muscle function in the LH and LH^17^LNa congenic rats would be necessary to determine if muscle wasting was occurring (Baek et al., 2020), and whether male hormones impacted the outcome in this model.

Ercc6l2 is a DNA repair enzyme involved in nucleotide excision repair (NER) and the nature of the mutation in the LH^17^LNa rats likely renders this protein completely nonfunctional, so future work will examine the role of this gene in DNA damage and oxidative stress sensitivity. Because Bone Marrow Failure Syndrome (BMF) has a known genetic cause, but the pathophysiology of the disease is unknown, future work will assess the clinical relevance of the Ercc6l2 dysfunction in the rat and investigate whether our rat model is a suitable model for human BMF, including inquiries into DNA damage and sensitivity to genotoxic stressors, such as chemotherapeutics. Because the TC-NER pathway has several redundancies, it will also be important to determine what DNA repair mechanisms might be upregulated or optimized in the LH^17^LNa rats to compensate for congenital Ercc6l2 deficiencies, and whether any of these pathways might be exploited to aid in clinical treatments for other DNA repair failure syndromes.

Reactive oxygen species (ROS) are necessary metabolic byproducts with important signaling functions in lipolysis and lipogenesis in the adipocyte (Abou-Rjeileh and Contreras, 2021), however, ROS are an intrinsic source of damage to genomic DNA (Turgeon et al., 2018), which can activate the TC-NER pathway (Lans et al., 2019). From our studies, we conclude that the newly discovered truncating mutation in *Ercc6l2* was the single best candidate underlying the phenotypes we observed in our novel congenic strain LH^17^LNa. Although the physiological relevance of this mutation is yet to be elucidated, DNA repair dysfunctions have broad clinical applications for greater understanding of neurological diseases, premature aging, and many other age-related diseases.

## Data Availability

The .vcf and BAM files for this study can be found in the Rat Genome Database at https://download.rgd.mcw.edu/strain_specific_variants/Dwinell_MCW_HybridRatDiversityProgram/. All genotyping and gene expression primers and probe sequences are available in the Supplementary Material.

## References

[B1] Abou-Rjeileh U. Contreras G. A. (2021). Redox Regulation of Lipid Mobilization in Adipose Tissues. Antioxidants (Basel) 10, 1090. 10.3390/antiox10071090 34356323 PMC8301038

[B2] Atanur S. S. Diaz A. G. Maratou K. Sarkis A. Rotival M. Game L. (2013). Genome Sequencing Reveals Loci under Artificial Selection that Underlie Disease Phenotypes in the Laboratory Rat. Cell 154, 691–703. 10.1016/j.cell.2013.06.040 23890820 PMC3732391

[B3] Baek K.-W. Jung Y.-K. Kim J.-S. Park J. S. Hah Y.-S. Kim S.-J. (2020). Rodent Model of Muscular Atrophy for Sarcopenia Study. J. Bone Metab. 27, 97–110. 10.11005/jbm.2020.27.2.97 32572370 PMC7297619

[B4] Banerjee D. Mandal S. M. Das A. Hegde M. L. Das S. Bhakat K. K. (2011). Preferential Repair of Oxidized Base Damage in the Transcribed Genes of Mammalian Cells. J. Biol. Chem. 286, 6006–6016. 10.1074/jbc.m110.198796 21169365 PMC3057786

[B5] Bierhuizen M. F. Mattei M. G. Fukuda M. (1993). Expression of the Developmental I Antigen by a Cloned Human cDNA Encoding a Member of a Beta-1,6-N-Acetylglucosaminyltransferase Gene Family. Genes Dev. 7, 468–478. 10.1101/gad.7.3.468 8449405

[B6] Bilusić M. Moreno C. Barreto N. E. Tschannen M. R. Harris E. L. Porteous W. K. (2008). Genetically Hypertensive Brown Norway Congenic Rat Strains Suggest Intermediate Traits Underlying Genetic Hypertension. Croat. Med. J. 49, 586–599. 10.3325/cmj.2008.5.586 18925692 PMC2582351

[B7] Bilusic M. Bataillard A. Tschannen M. R. Gao L. Barreto N. E. Vincent M. (2004). Mapping the Genetic Determinants of Hypertension, Metabolic Diseases, and Related Phenotypes in the Lyon Hypertensive Rat. Hypertension 44, 695–701. 10.1161/01.hyp.0000144542.57306.5e 15452030

[B8] Blüher M. (2012). Clinical Relevance of Adipokines. Diabetes Metab. J. 36, 317–327. 10.4093/dmj.2012.36.5.317 23130315 PMC3486977

[B9] Brace L. E. Vose S. C. Vargas D. F. Zhao S. Wang X.-P. Mitchell J. R. (2013). Lifespan Extension by Dietary Intervention in a Mouse Model of Cockayne Syndrome Uncouples Early Postnatal Development from Segmental Progeria. Aging Cell 12, 1144–1147. 10.1111/acel.12142 23895664 PMC4266594

[B10] Brandkvist M. Bjørngaard J. H. Ødegård R. A. Åsvold B. O. Sund E. R. Vie G. Å. (2019). Quantifying the Impact of Genes on Body Mass Index during the Obesity Epidemic: Longitudinal Findings from the HUNT Study. BMJ 366, l4067. 10.1136/bmj.l4067 31270083 PMC6607203

[B11] Cai L. Wang Z. Ji A. Meyer J. M. van der Westhuyzen D. R. (2012). Scavenger Receptor CD36 Expression Contributes to Adipose Tissue Inflammation and Cell Death in Diet-Induced Obesity. PLoS One 7, e36785. 10.1371/journal.pone.0036785 22615812 PMC3353961

[B12] Choi Y. Chan A. P. (2015). PROVEAN Web Server: a Tool to Predict the Functional Effect of Amino Acid Substitutions and Indels. Bioinformatics 31, 2745–2747. 10.1093/bioinformatics/btv195 25851949 PMC4528627

[B13] Cingolani P. Platts A. Wang L. L. Coon M. Nguyen T. Wang L. (2012). A Program for Annotating and Predicting the Effects of Single Nucleotide Polymorphisms, SnpEff: SNPs in the Genome of *Drosophila melanogaster* Strain W1118; Iso-2; Iso-3. Fly 6, 80–92. 10.4161/fly.19695 22728672 PMC3679285

[B14] Clark K. C. Kwitek A. E. (2021). Multi‐Omic Approaches to Identify Genetic Factors in Metabolic Syndrome. Compr. Physiol. 12, 3045–3084. 10.1002/cphy.c210010 34964118 PMC9373910

[B15] Dimitroff C. J. (2019). I-branched Carbohydrates as Emerging Effectors of Malignant Progression. Proc. Natl. Acad. Sci. U.S.A. 116, 13729–13737. 10.1073/pnas.1900268116 31213534 PMC6628663

[B16] Gilibert S. Bataillard A. Nussberger J. Sassard J. Kwitek A. E. (2009). Implication of Chromosome 13 on Hypertension and Associated Disorders in Lyon Hypertensive Rats. J. Hypertens. 27, 1186–1193. 10.1097/hjh.0b013e328329e4c0 19462495 PMC2915542

[B17] Gilibert S. Kwitek A. E. Hubner N. Tschannen M. Jacob H. J. Sassard J. (2008). Effects of Chromosome 17 on Features of the Metabolic Syndrome in the Lyon Hypertensive Rat. Physiol. Genomics 33, 212–217. 10.1152/physiolgenomics.00262.2007 18285521

[B18] Gonzales A. M. Orlando R. A. (2007). Role of Adipocyte-Derived Lipoprotein Lipase in Adipocyte Hypertrophy. Nutr. Metab. (Lond) 4, 22. 10.1186/1743-7075-4-22 17971230 PMC2174487

[B19] Grobe J. L. (2017). Comprehensive Assessments of Energy Balance in Mice. Methods Mol. Biol. 1614, 123–146. 10.1007/978-1-4939-7030-8_10 28500600 PMC5582947

[B20] Halberg N. Khan T. Trujillo M. E. Wernstedt-Asterholm I. Attie A. D. Sherwani S. (2009). Hypoxia-Inducible Factor 1α Induces Fibrosis and Insulin Resistance in White Adipose Tissue. Mol. Cell Biol. 29, 4467–4483. 10.1128/mcb.00192-09 19546236 PMC2725728

[B21] Howe K. Dwinell M. Shimoyama M. Corton C. Betteridge E. Dove A. (2021). The Genome Sequence of the Norway Rat, *Rattus norvegicus* Berkenhout 1769. Wellcome Open Res. 6, 118. 10.12688/wellcomeopenres.16854.1 34660910 PMC8495504

[B22] Inaba N. Hiruma T. Togayachi A. Iwasaki H. Wang X.-H. Furukawa Y. (2003). A Novel I-Branching β-1,6-N-acetylglucosaminyltransferase Involved in Human Blood Group I Antigen Expression. Blood 101, 2870–2876. 10.1182/blood-2002-09-2838 12468428

[B23] Jarviaho T. Halt K. Hirvikoski P. Moilanen J. Möttönen M. Niinimäki R. (2018). Bone Marrow Failure Syndrome Caused by Homozygous Frameshift Mutation in the ERCC6L2 Gene. Clin. Genet. 93, 392–395. 10.1111/cge.13125 28815563

[B24] Kwitek A. E. (2019). Rat Models of Metabolic Syndrome. Methods Mol. Biol. 2018, 269–285. 10.1007/978-1-4939-9581-3_13 31228162 PMC7315404

[B25] Lans H. Hoeijmakers J. H. J. Vermeulen W. Marteijn J. A. (2019). The DNA Damage Response to Transcription Stress. Nat. Rev. Mol. Cell Biol. 20, 766–784. 10.1038/s41580-019-0169-4 31558824

[B26] Liu X. Liu T. Shang Y. Dai P. Zhang W. Lee B. J. (2020). ERCC6L2 Promotes DNA Orientation-specific Recombination in Mammalian Cells. Cell Res. 30, 732–744. 10.1038/s41422-020-0328-3 32355287 PMC7608219

[B27] Livak K. J. Schmittgen T. D. (2001). Analysis of Relative Gene Expression Data Using Real-Time Quantitative PCR and the 2−ΔΔCT Method. Methods 25, 402–408. 10.1006/meth.2001.1262 11846609

[B28] Ma M. C. J. Atanur S. S. Aitman T. J. Kwitek A. E. (2014). Genomic Structure of Nucleotide Diversity Among Lyon Rat Models of Metabolic Syndrome. BMC Genomics 15, 197. 10.1186/1471-2164-15-197 24628878 PMC4003853

[B29] Ma M. C. J. Pettus J. M. Jakoubek J. A. Traxler M. G. Clark K. C. Mennie A. K. (2017). Contribution of Independent and Pleiotropic Genetic Effects in the Metabolic Syndrome in a Hypertensive Rat. PLoS One 12, e0182650. 10.1371/journal.pone.0182650 28792545 PMC5549746

[B30] Martín-Gálvez D. Dunoyer de Segonzac D. Ma M. C. J. Kwitek A. E. Thybert D. Flicek P. (2017). Genome Variation and Conserved Regulation Identify Genomic Regions Responsible for Strain Specific Phenotypes in Rat. BMC Genomics 18, 986. 10.1186/s12864-017-4351-9 29272997 PMC5741965

[B31] Mertes F. Martinez-Morales J.-R. Nolden T. Spörle R. Wittbrodt J. Lehrach H. (2009). Cloning of Mouse Ojoplano, a Reticular Cytoplasmic Protein Expressed during Embryonic Development. Gene Expr. Patterns 9, 562–567. 10.1016/j.gep.2009.09.003 19766735

[B32] Mikami J. Tobisawa Y. Yoneyama T. Hatakeyama S. Mori K. Hashimoto Y. (2016). I-branching N-Acetylglucosaminyltransferase Regulates Prostate Cancer Invasiveness by Enhancing Alpha5beta1 Integrin Signaling. Cancer Sci. 107, 359–368. 10.1111/cas.12859 26678556 PMC4814258

[B33] Moreno-Indias I. Tinahones F. J. (2015). Impaired Adipose Tissue Expandability and Lipogenic Capacities as Ones of the Main Causes of Metabolic Disorders. J. Diabetes Res. 2015, 970375. 10.1155/2015/970375 25922847 PMC4398959

[B34] Nakazawa Y. Hara Y. Oka Y. Komine O. van Den Heuvel D. Guo C. (2020). Ubiquitination of DNA Damage-Stalled RNAPII Promotes Transcription-Coupled Repair. Cell 180, 1228–1244. 10.1016/j.cell.2020.02.010 32142649

[B36] Ohnishi T. Yamada K. Watanabe A. Ohba H. Sakaguchi T. Honma Y. (2011). Ablation of Mrds1/Ofcc1 Induces Hyper-Gamma-Glutamyl Transpeptidasemia without Abnormal Head Development and Schizophrenia-Relevant Behaviors in Mice. PLoS One 6, e29499. 10.1371/journal.pone.0029499 22242126 PMC3248446

[B37] Peng F. He Q. Cheng C. Pan J. (2019). GCNT2 Induces Epithelial-Mesenchymal Transition and Promotes Migration and Invasion in Esophageal Squamous Cell Carcinoma Cells. Cell Biochem. Funct. 37, 42–51. 10.1002/cbf.3371 30575058

[B38] Reho J. J. Nakagawa P. Mouradian G. C. JR. Grobe C. C. Saravia F. L. Burnett C. M. L. (2022). Methods for the Comprehensive *In Vivo* Analysis of Energy Flux, Fluid Homeostasis, Blood Pressure, and Ventilatory Function in Rodents. Front. Physiol. 13, 855054. 10.3389/fphys.2022.855054 35283781 PMC8914175

[B39] Roh E. Choi K. M. (2020). Health Consequences of Sarcopenic Obesity: A Narrative Review. Front. Endocrinol. 11, 332. 10.3389/fendo.2020.00332 PMC7253580 32508753

[B40] Ryan D. P. Owen-Hughes T. (2011). Snf2-family Proteins: Chromatin Remodellers for Any Occasion. Curr. Opin. Chem. Biol. 15, 649–656. 10.1016/j.cbpa.2011.07.022 21862382 PMC4162295

[B41] Sassard J. Gilibert S. Bataillard A. (2007). Consomic Approach to Blood Pressure and Metabolic Diseases in Lyon Hypertensive Rats. Bull. l'Acad. Natl. Méd. 191, 849–856. discussion 855-6. 10.1016/s0001-4079(19)33022-5 18225439

[B42] Sassolas A. Vincent M. Benzoni D. Sassard J. (1981). Plasma Lipids in Genetically Hypertensive Rats of the Lyon Strain. J. Cardiovasc. Pharmacol. 3, 1008–1014. 10.1097/00005344-198109000-00011 6168846

[B43] Shabanova I. Cohen E. Cada M. Vincent A. Cohn R. D. Dror Y. (2018). ERCC6L2 -associated Inherited Bone Marrow Failure Syndrome. Mol. Genet. Genomic Med. 6, 463–468. 10.1002/mgg3.388 29633571 PMC6014454

[B44] Smith J. R. Hayman G. T. Wang S. J. Laulederkind S. J. F. Hoffman M. J. Kaldunski M. L. (2020). The Year of the Rat: The Rat Genome Database at 20: a Multi-Species Knowledgebase and Analysis Platform. Nucleic Acids Res. 48, D731–D742. 10.1093/nar/gkz1041 31713623 PMC7145519

[B45] Tummala H. Dokal A. D. Walne A. Ellison A. Cardoso S. Amirthasigamanipillai S. (2018). Genome Instability Is a Consequence of Transcription Deficiency in Patients with Bone Marrow Failure Harboring Biallelic ERCC6L2 Variants. Proc. Natl. Acad. Sci. U.S.A. 115, 7777–7782. 10.1073/pnas.1803275115 29987015 PMC6064997

[B46] Tummala H. Kirwan M. Walne A. J. Hossain U. Jackson N. Pondarre C. (2014). ERCC6L2 Mutations Link a Distinct Bone-Marrow-Failure Syndrome to DNA Repair and Mitochondrial Function. Am. J. Hum. Genet. 94, 246–256. 10.1016/j.ajhg.2014.01.007 24507776 PMC3928664

[B47] Turgeon M.-O. Perry N. J. S. Poulogiannis G. (2018). DNA Damage, Repair, and Cancer Metabolism. Front. Oncol. 8, 15. 10.3389/fonc.2018.00015 29459886 PMC5807667

[B48] Tutaj M. Smith J. R. Bolton E. R. (2019). Rat Genome Assemblies, Annotation, and Variant Repository. Methods Mol. Biol. 2018, 43–70. 10.1007/978-1-4939-9581-3_2 31228151

[B49] van der Klaauw A. A. Farooqi I. S. (2015). The Hunger Genes: Pathways to Obesity. Cell 161, 119–132. 10.1016/j.cell.2015.03.008 25815990

[B50] Vermeij W. P. Dollé M. E. T. Reiling E. Jaarsma D. Payan-Gomez C. Bombardieri C. R. (2016). Restricted Diet Delays Accelerated Ageing and Genomic Stress in DNA-Repair-Deficient Mice. Nature 537, 427–431. 10.1038/nature19329 27556946 PMC5161687

[B51] Vincent M. Samani N. J. Gauguier D. Thompson J. R. Lathrop G. M. Sassard J. (1997). A Pharmacogenetic Approach to Blood Pressure in Lyon Hypertensive Rats. A Chromosome 2 Locus Influences the Response to a Calcium Antagonist. J. Clin. Invest. 100, 2000–2006. 10.1172/jci119731 9329963 PMC508389

[B52] Wakeland E. Morel L. Achey K. Yui M. Longmate J. (1997). Speed Congenics: a Classic Technique in the Fast Lane (Relatively Speaking). Immunol. Today 18, 472–477. 10.1016/s0167-5699(97)01126-2 9357138

[B53] Wang J. Ma M. C. J. Mennie A. K. Pettus J. M. Xu Y. Lin L. (2015). Systems Biology with High-Throughput Sequencing Reveals Genetic Mechanisms Underlying the Metabolic Syndrome in the Lyon Hypertensive Rat. Circ. Cardiovasc Genet. 8, 316–326. 10.1161/circgenetics.114.000520 25573024 PMC4406788

[B54] Wardle J. Carnell S. Haworth C. M. Plomin R. (2008). Evidence for a Strong Genetic Influence on Childhood Adiposity Despite the Force of the Obesogenic Environment. Am. J. Clin. Nutr. 87, 398–404. 10.1093/ajcn/87.2.398 18258631

[B55] Ye R. Z. Richard G. Gévry N. Tchernof A. Carpentier A. C. (2022). Fat Cell Size: Measurement Methods, Pathophysiological Origins, and Relationships with Metabolic Dysregulations. Endocr. Rev. 43, 35–60. 10.1210/endrev/bnab018 34100954 PMC8755996

[B56] Yu L.-C. Twu Y.-C. Chou M.-L. Reid M. E. Gray A. R. Moulds J. M. (2003). The Molecular Genetics of the Human I Locus and Molecular Background Explain the Partial Association of the Adult I Phenotype with Congenital Cataracts. Blood 101, 2081–2087. 10.1182/blood-2002-09-2693 12424189

[B57] Zhang S. Pondarre C. Pennarun G. Labussiere-Wallet H. Vera G. France B. (2016). A Nonsense Mutation in the DNA Repair Factor Hebo Causes Mild Bone Marrow Failure and Microcephaly. J. Exp. Med. 213, 1011–1028. 10.1084/jem.20151183 27185855 PMC4886357

