# Supplementary material for: Body Composition and Metabolic Changes in a Lyon Hypertensive Congenic Rat and Identification of Ercc6l2 as a Positional Candidate Gene
Source: Front Genet. 2022 Jun 24;13:903971. doi: 10.3389/fgene.2022.903971 (PMC9263446; doi:10.3389/fgene.2022.903971)
Supplement: Supplementary file 1 [file DataSheet2.PDF]

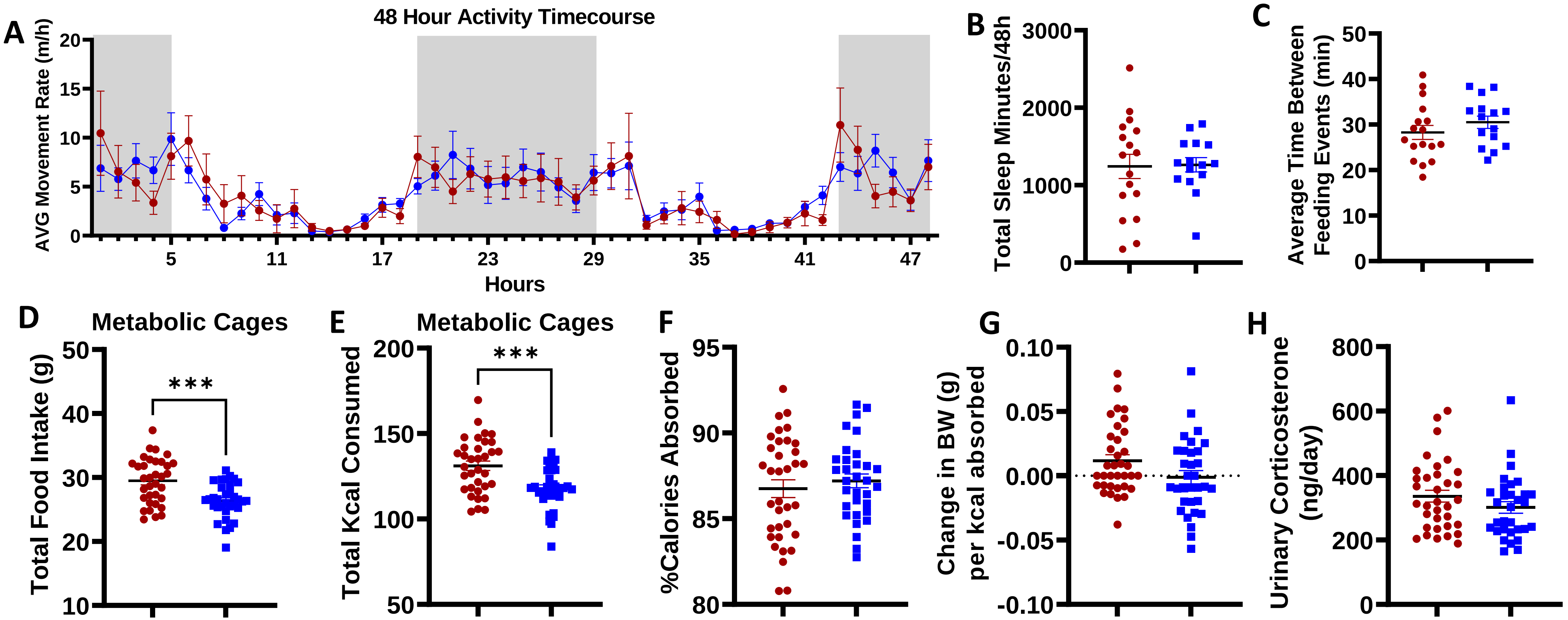

Supplemental Figure 2: Additional energy balance data in LH^{17}LNa female congenic rats

(A) LH^{17}LNa congenic females displayed no difference in activity (shown as hourly average rate per animal, plotted over 48 hours) (LH: n=11; LH^{17}LNa: n=14). **(B)** LH^{17}LNa females displayed no differences in total time spent sleeping (LH: n=17; LH^{17}LNa: n=15). **(C)** No differences were found between the LH (n=17) and LH^{17}LNa females (n=15) for average time between meals in minutes. In single metabolic cage setups between LH (n=34) and LH^{17}LNa (n=33) females, both **(D)** food intake and **(E)** total kcal consumed were decreased (p<0.001, unpaired, two-tailed t-test). No differences in **(F)** digestive efficiency or **(F)** energy efficiency were noted between LH control females (n=35) and LH^{17}LNa females (n=33) during the final 48 hours in metabolic caging. No significant difference in **(H)** total urinary corticosterone (ng) elimination per day was seen between LH controls (n=34) and LH^{17}LNa females (n=31).
