# Supplementary figures and images for: Body Composition and Metabolic Changes in a Lyon Hypertensive Congenic Rat and Identification of Ercc6l2 as a Positional Candidate Gene
Source: Front Genet. 2022 Jun 24;13:903971. doi: 10.3389/fgene.2022.903971 (PMC9263446; doi:10.3389/fgene.2022.903971)

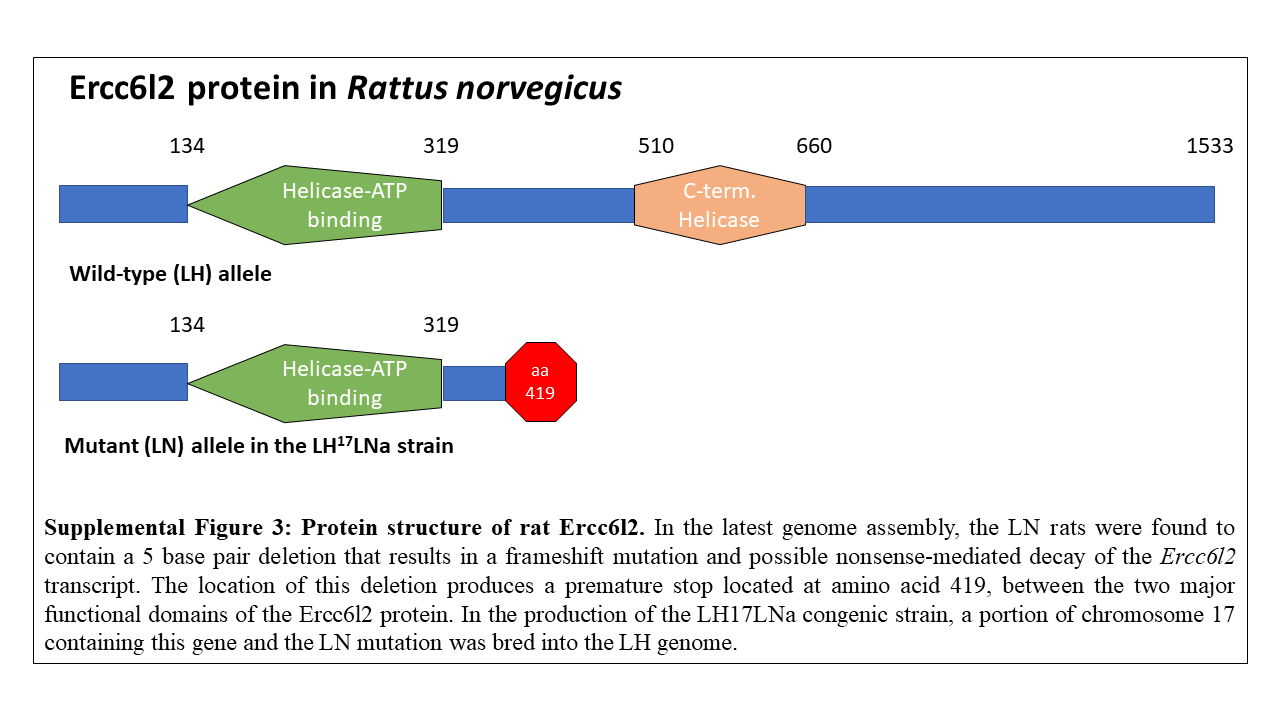

Supplement: Supplementary file 2 [file Image1.TIF]
