# Supplementary material for: Body Composition and Metabolic Changes in a Lyon Hypertensive Congenic Rat and Identification of Ercc6l2 as a Positional Candidate Gene
Source: Front Genet. 2022 Jun 24;13:903971. doi: 10.3389/fgene.2022.903971 (PMC9263446; doi:10.3389/fgene.2022.903971)
Supplement: Supplementary file 3 [file DataSheet1.PDF]

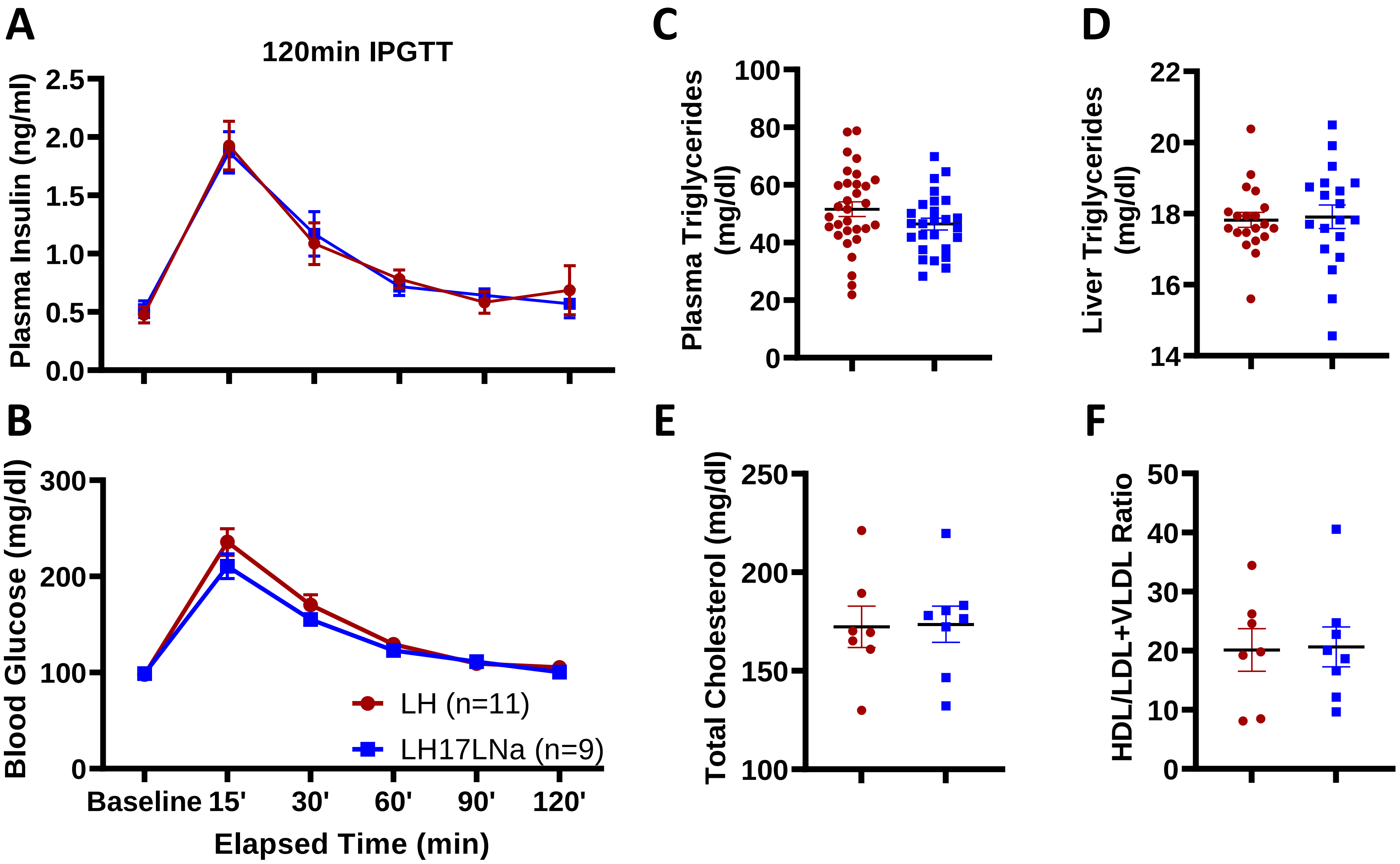

Supplemental Figure 1: Other MetS features do not differ between the LH and LH^{17}LNa strains

(A-B) Glucose tolerance tests (IPGTT) in LH (red circle) and LH^{17}LNa (blue square) female rats were not different at indicated time points after dextrose injections (1g/kg; IP) (plasma insulin: LH n=9, LH^{17}LNa n=8; blood glucose: LH n=11, LH^{17}LNa n=9). No differences were observed in **(C)** plasma triglycerides (LH: n=31; LH^{17}LNa: n=26), or in triglycerides isolated from **(D)** liver homogenates (LH: n=20; LH^{17}LNa: n=19). **(E-F)** Total serum cholesterol and HDL- and LDL-cholesterol fractions were measured in serum (LH: n=7, LH^{17}LNa: n=8), and no differences were seen in either total serum cholesterol **(E)**, or in HDL/LDL cholesterol ratio **(F)**.
